# Nano based drug delivery systems: recent developments and future prospects

**DOI:** 10.1186/s12951-018-0392-8

**Published:** 2018-09-19

**Authors:** Jayanta Kumar Patra, Gitishree Das, Leonardo Fernandes Fraceto, Estefania Vangelie Ramos Campos, Maria del Pilar Rodriguez-Torres, Laura Susana Acosta-Torres, Luis Armando Diaz-Torres, Renato Grillo, Mallappa Kumara Swamy, Shivesh Sharma, Solomon Habtemariam, Han-Seung Shin

**Affiliations:** 1 Research Institute of Biotechnology & Medical Converged Science, Dongguk University-Seoul, Goyang-si, 10326 Republic of Korea; 2 Sao Paulo State University (UNESP), Institute of Science and Technology, Sorocaba, São Paulo Zip Code 18087-180 Brazil; 3 Department of Biochemistry and Tissue Biology, Institute of Biology, State University of Campinas, Campinas, São Paulo Zip code 13083-862 Brazil; 4 Laboratorio de Investigación Interdisciplinaria, Área de Nanoestructuras y Biomateriales, Escuela Nacional de Estudios Superiores, Unidad Leon, Universidad Nacional Autonóma de México (UNAM), Boulevard UNAM No 2011. Predio El Saucillo y El Potrero, 37684 León, Guanajuato Mexico; 5 Centro de Investigaciones en Óptica, A.P. 1-948, C.P. 37000 León, Guanajuato Mexico; 6 Department of Physics and Chemistry, School of Engineering, São Paulo State University (UNESP), Ilha Solteira, SP 15385-000 Brazil; 7 Department of Crop Science, Faculty of Agriculture, Universiti Putra Malaysia, 43400 Serdang, Selangor Malaysia; 8 Department of Biotechnology, Motilal Nehru National Institute of Technology Allahabad, Allahabad, Uttar Pradesh 211004 India; 9 Pharmacognosy Research Laboratories & Herbal Analysis Services UK, University of Greenwich, Medway Campus-Science, Grenville Building (G102/G107), Central Avenue, Chatham-Maritime, Kent, ME4 4TB UK; 10 Department of Food Science and Biotechnology, Dongguk University, Ilsandong-gu, Goyang, Gyeonggi-do 10326 Republic of Korea

**Keywords:** Nanomedicine, Nanomaterials, Drug delivery, Drug targeting, Natural products

## Abstract

Nanomedicine and nano delivery systems are a relatively new but rapidly developing science where materials in the nanoscale range are employed to serve as means of diagnostic tools or to deliver therapeutic agents to specific targeted sites in a controlled manner. Nanotechnology offers multiple benefits in treating chronic human diseases by site-specific, and target-oriented delivery of precise medicines. Recently, there are a number of outstanding applications of the nanomedicine (chemotherapeutic agents, biological agents, immunotherapeutic agents etc.) in the treatment of various diseases. The current review, presents an updated summary of recent advances in the field of nanomedicines and nano based drug delivery systems through comprehensive scrutiny of the discovery and application of nanomaterials in improving both the efficacy of novel and old drugs (e.g., natural products) and selective diagnosis through disease marker molecules. The opportunities and challenges of nanomedicines in drug delivery from synthetic/natural sources to their clinical applications are also discussed. In addition, we have included information regarding the trends and perspectives in nanomedicine area.

## Background

Since ancient times, humans have widely used plant-based natural products as medicines against various diseases. Modern medicines are mainly derived from herbs on the basis of traditional knowledge and practices. Nearly, 25% of the major pharmaceutical compounds and their derivatives available today are obtained from natural resources [1, 2]. Natural compounds with different molecular backgrounds present a basis for the discovery of novel drugs. A recent trend in the natural product-based drug discovery has been the interest in designing synthetically amenable lead molecules, which mimic their counterpart’s chemistry [3]. Natural products exhibit remarkable characteristics such as extraordinary chemical diversity, chemical and biological properties with macromolecular specificity and less toxicity. These make them favorable leads in the discovery of novel drugs [4]. Further, computational studies have helped envisage molecular interactions of drugs and develop next-generation drug inventions such as target-based drug discovery and drug delivery.

Despite several advantages, pharmaceutical companies are hesitant to invest more in natural product-based drug discovery and drug delivery systems [5] and instead explore the available chemical compounds libraries to discover novel drugs. However, natural compounds are now being screened for treating several major diseases, including cancer, diabetes, cardiovascular, inflammatory, and microbial diseases. This is mainly because natural drugs possess unique advantages, such as lower toxicity and side effects, low-price, and good therapeutic potential. However, concerns associated with the biocompatibility, and toxicity of natural compounds presents a greater challenge of using them as medicine. Consequently, many natural compounds are not clearing the clinical trial phases because of these problems [6–8]. The use of large sized materials in drug delivery poses major challenges, including in vivo instability, poor bioavailability, and poor solubility, poor absorption in the body, issues with target-specific delivery, and tonic effectiveness, and probable adverse effects of drugs. Therefore, using new drug delivery systems for targeting drugs to specific body parts could be an option that might solve these critical issues [9, 10]. Hence, nanotechnology plays a significant role in advanced medicine/drug formulations, targeting arena and their controlled drug release and delivery with immense success.

**Fig. 1:**
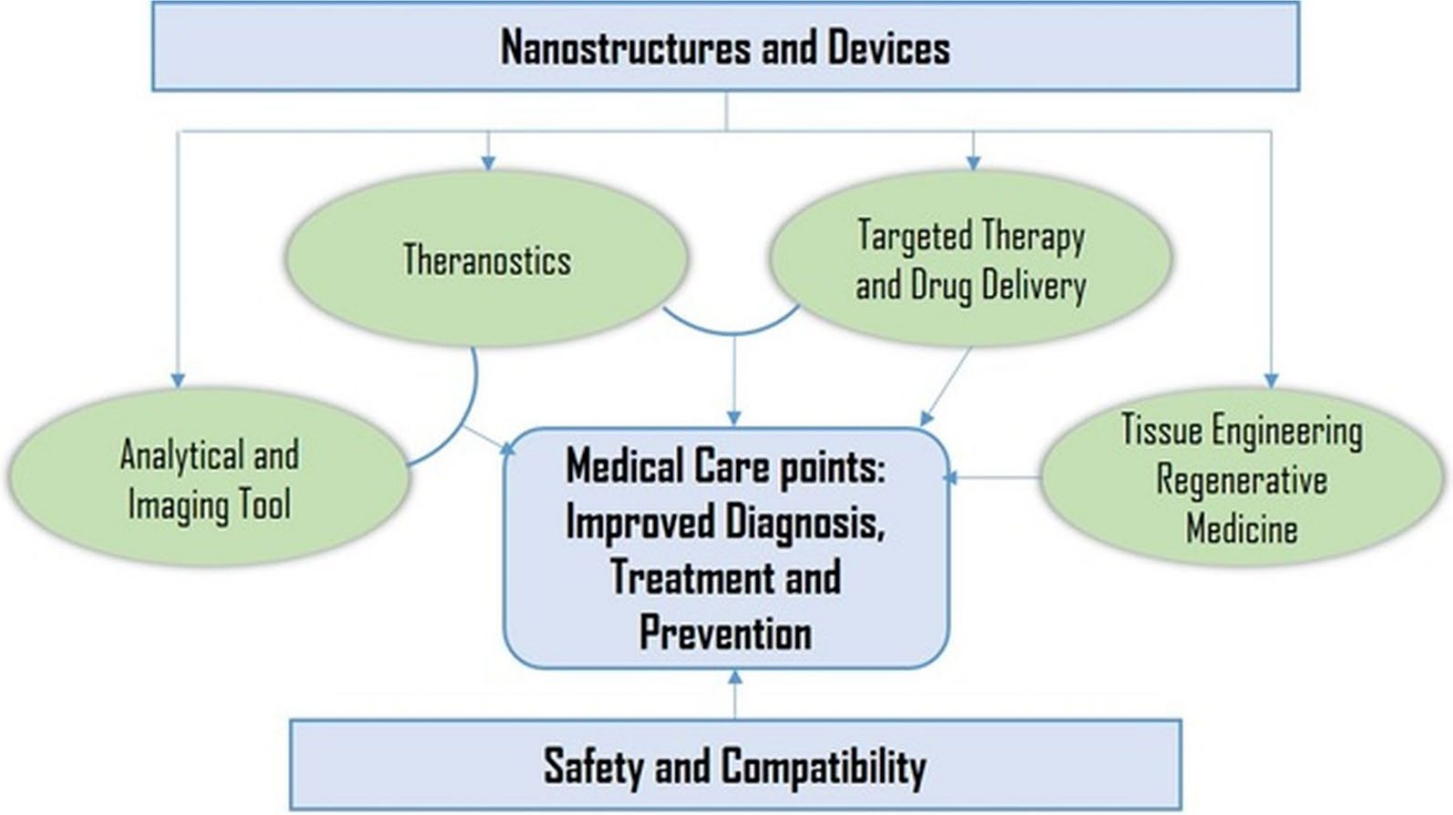
Application and goals of nanomedicine in different sphere of biomedical research

Nanotechnology is shown to bridge the barrier of biological and physical sciences by applying nanostructures and nanophases at various fields of science [11]; specially in nanomedicine and nano based drug delivery systems, where such particles are of major interest [12, 13]. Nanomaterials can be well-defined as a material with sizes ranged between 1 and 100 nm, which influences the frontiers of nanomedicine starting from biosensors, microfluidics, drug delivery, and microarray tests to tissue engineering [14–16]. Nanotechnology employs curative agents at the nanoscale level to develop nanomedicines. The field of biomedicine comprising nanobiotechnology, drug delivery, biosensors, and tissue engineering has been powered by nanoparticles [17]. As nanoparticles comprise materials designed at the atomic or molecular level, they are usually small sized nanospheres [18]. Hence, they can move more freely in the human body as compared to bigger materials. Nanoscale sized particles exhibit unique structural, chemical, mechanical, magnetic, electrical, and biological properties. Nanomedicines have become well appreciated in recent times due to the fact that nanostructures could be utilized as delivery agents by encapsulating drugs or attaching therapeutic drugs and deliver them to target tissues more precisely with a controlled release [10, 19]. Nanomedicine, is an emerging field implementing the use of knowledge and techniques of nanoscience in medical biology and disease prevention and remediation. It implicates the utilization of nanodimensional materials including nanorobots, nanosensors for diagnosis, delivery, and sensory purposes, and actuate materials in live cells (Fig. 1). For example, a nanoparticle-based method has been developed which combined both the treatment and imaging modalities of cancer diagnosis [20]. The very first generation of nanoparticle-based therapy included lipid systems like liposomes and micelles, which are now FDA-approved [21]. These liposomes and micelles can contain inorganic nanoparticles like gold or magnetic nanoparticles [22]. These properties let to an increase in the use of inorganic nanoparticles with an emphasis on drug delivery, imaging and therapeutics functions. In addition, nanostructures reportedly aid in preventing drugs from being tarnished in the gastrointestinal region and help the delivery of sparingly water-soluble drugs to their target location. Nanodrugs show higher oral bioavailability because they exhibit typical uptake mechanisms of absorptive endocytosis.

Nanostructures stay in the blood circulatory system for a prolonged period and enable the release of amalgamated drugs as per the specified dose. Thus, they cause fewer plasma fluctuations with reduced adverse effects [23]. Being nanosized, these structures penetrate in the tissue system, facilitate easy uptake of the drug by cells, permit an efficient drug delivery, and ensure action at the targeted location. The uptake of nanostructures by cells is much higher than that of large particles with size ranging between 1 and 10 µm [17, 24]. Hence, they directly interact to treat the diseased cells with improved efficiency and reduced or negligible side effects.

At all stages of clinical practices, nanoparticles have been found to be useful in acquiring information owing to their use in numerous novel assays to treat and diagnose diseases. The main benefits of these nanoparticles are associated with their surface properties; as various proteins can be affixed to the surface. For instance, gold nanoparticles are used as biomarkers and tumor labels for various biomolecule detection procedural assays.

Regarding the use of nanomaterials in drug delivery, the selection of the nanoparticle is based on the physicochemical features of drugs. The combined use of nanoscience along with bioactive natural compounds is very attractive, and growing very rapidly in recent times. It presents several advantages when it comes to the delivery of natural products for treating cancer and many other diseases. Natural compounds have been comprehensively studied in curing diseases owing to their various characteristic activities, such as inducing tumor-suppressing autophagy and acting as antimicrobial agents. Autophagy has been observed in curcumin and caffeine [25], whereas antimicrobial effects have been shown by cinnamaldehyde, carvacrol, curcumin and eugenol [26, 27]. The enrichment of their properties, such as bioavailability, targeting and controlled release were made by incorporating nanoparticles. For instance, thymoquinone, a bioactive compound in *Nigella sativa*, is studied after its encapsulation in lipid nanocarrier. After encapsulation, it showed sixfold increase in bioavailability in comparison to free thymoquinone and thus protects the gastrointestinal stuffs [28]. It also increased the pharmacokinetic characteristics of the natural product resulting in better therapeutic effects.

Metallic, organic, inorganic and polymeric nanostructures, including dendrimers, micelles, and liposomes are frequently considered in designing the target-specific drug delivery systems. In particular, those drugs having poor solubility with less absorption ability are tagged with these nanoparticles [17, 29]. However, the efficacy of these nanostructures as drug delivery vehicles varies depending on the size, shape, and other inherent biophysical/chemical characteristics. For instance, polymeric nanomaterials with diameters ranging from 10 to 1000 nm, exhibit characteristics ideal for an efficient delivery vehicle [7]. Because of their high biocompatibility and biodegradability properties, various synthetic polymers such as polyvinyl alcohol, poly-l-lactic acid, polyethylene glycol, and poly(lactic-*co*-glycolic acid), and natural polymers, such as alginate and chitosan, are extensively used in the nanofabrication of nanoparticles [8, 30–32]. Polymeric nanoparticles can be categorized into nanospheres and nanocapsules both of which are excellent drug delivery systems. Likewise, compact lipid nanostructures and phospholipids including liposomes and micelles are very useful in targeted drug delivery.

The use of ideal nano-drug delivery system is decided primarily based on the biophysical and biochemical properties of the targeted drugs being selected for the treatment [8]. However, problems such as toxicity exhibited by nanoparticles cannot be ignored when considering the use of nanomedicine. More recently, nanoparticles have mostly been used in combination with natural products to lower the toxicity issues. The green chemistry route of designing nanoparticles loaded with drugs is widely encouraged as it minimises the hazardous constituents in the biosynthetic process. Thus, using green nanoparticles for drug delivery can lessen the side-effects of the medications [19]. Moreover, adjustments in nanostructures size, shape, hydrophobicity, and surface changes can further enhance the bioactivity of these nanomaterials.

Thus, nanotechnology offers multiple benefits in treating chronic human diseases by site-specific, and target-oriented delivery of medicines. However, inadequate knowledge about nanostructures toxicity is a major worry and undoubtedly warrants further research to improve the efficacy with higher safety to enable safer practical implementation of these medicines. Therefore, cautiously designing these nanoparticles could be helpful in tackling the problems associated with their use. Considering the above facts, this review aims to report different nano based drug delivery systems, significant applications of natural compound-based nanomedicines, and bioavailability, targeting sites, and controlled release of nano-drugs, as well as other challenges associated with nanomaterials in medicines.

## Nano based drug delivery systems

Recently, there has been enormous developments in the field of delivery systems to provide therapeutic agents or natural based active compounds to its target location for treatment of various aliments [33, 34]. There are a number of drug delivery systems successfully employed in the recent times, however there are still certain challenges that need to be addresses and an advanced technology need to be developed for successful delivery of drugs to its target sites. Hence the nano based drug delivery systems are currently been studied that will facilitate the advanced system of drug delivery.

### Fundamentals of nanotechnology based techniques in designing of drug

Nanomedicine is the branch of medicine that utilizes the science of nanotechnology in the preclusion and cure of various diseases using the nanoscale materials, such as biocompatible nanoparticles [35] and nanorobots [36], for various applications including, diagnosis [37], delivery [38], sensory [39], or actuation purposes in a living organism [40]. Drugs with very low solubility possess various biopharmaceutical delivery issues including limited bio accessibility after intake through mouth, less diffusion capacity into the outer membrane, require more quantity for intravenous intake and unwanted after-effects preceding traditional formulated vaccination process. However all these limitations could be overcome by the application of nanotechnology approaches in the drug delivery mechanism.

Drug designing at the nanoscale has been studied extensively and is by far, the most advanced technology in the area of nanoparticle applications because of its potential advantages such as the possibility to modify properties like solubility, drug release profiles, diffusivity, bioavailability and immunogenicity. This, can consequently lead to the improvement and development of convenient administration routes, lower toxicity, fewer side effects, improved biodistribution and extended drug life cycle [17]. The engineered drug delivery systems are either targeted to a particular location or are intended for the controlled release of therapeutic agents at a particular site. Their formation involves self-assembly where in well-defined structures or patterns spontaneously are formed from building blocks [41]. Additionally they need to overcome barriers like opsonization/sequestration by the mononuclear phagocyte system [42].

There are two ways through which nanostructures deliver drugs: passive and self-delivery. In the former, drugs are incorporated in the inner cavity of the structure mainly via the hydrophobic effect. When the nanostructure materials are targeted to a particular sites, the intended amount of the drug is released because of the low content of the drugs which is encapsulated in a hydrophobic environment [41]. Conversely, in the latter, the drugs intended for release are directly conjugated to the carrier nanostructure material for easy delivery. In this approach, the timing of release is crucial as the drug will not reach the target site and it dissociates from the carrier very quickly, and conversely, its bioactivity and efficacy will be decreased if it is released from its nanocarrier system at the right time [41]. Targeting of drugs is another significant aspect that uses nanomaterials or nanoformulations as the drug delivery systems and, is classified into active and passive. In active targeting, moieties, such as antibodies and peptides are coupled with drug delivery system to anchor them to the receptor structures expressed at the target site. In passive targeting, the prepared drug carrier complex circulates through the bloodstream and is driven to the target site by affinity or binding influenced by properties like pH, temperature, molecular site and shape. The main targets in the body are the receptors on cell membranes, lipid components of the cell membrane and antigens or proteins on the cell surfaces [43]. Currently, most nanotechnology-mediated drug delivery system are targeted towards the cancer disease and its cure.

### Biopolymeric nanoparticles in diagnosis, detection and imaging

The integration of therapy and diagnosis is defined as theranostic and is being extensively utilized for cancer treatment [44, 45]. Theranostic nanoparticles can help diagnose the disease, report the location, identify the stage of the disease, and provide information about the treatment response. In addition, such nanoparticles can carry a therapeutic agent for the tumor, which can provide the necessary concentrations of the therapeutic agent via molecular and/or external stimuli [44, 45]. Chitosan is a biopolymer which possesses distinctive properties with biocompatibility and presence of functional groups [45–47]. It is used in the encapsulation or coating of various types of nanoparticles, thus producing different particles with multiple functions for their potential uses in the detection and diagnosis of different types of diseases [45, 47].

Lee et al. [48] encapsulated oleic acid-coated FeO nanoparticles in oleic acid-conjugated chitosan (oleyl-chitosan) to examine the accretion of these nanoparticles in tumor cells through the penetrability and holding (EPR) consequence under the in vivo state for analytical uses by the near-infrared and magnetic resonance imaging (MRI) mechanisms. By the in vivo evaluations, both techniques showed noticeable signal strength and improvement in the tumor tissues through a higher EPR consequence after the injection of cyanine-5-attached oleyl-chitosan nanoparticles intravenously (Cyanine 5).

Yang et al. [49] prepared highly effective nanoparticles for revealing colorectal cancer (CC) cells via a light-mediated mechanism; these cells are visible owing to the physical conjugation of alginate with folic acid-modified chitosan leading to the formation of nanoparticles with enhanced 5-aminolevulinic (5-ALA) release in the cell lysosome. The results displayed that the engineered nanoparticles were voluntarily endocytosed by the CC cells by the folate receptor-based endocytosis process. Subsequently, the charged 5-ALA was dispersed into the lysosome which was triggered by less desirability strength between the 5-ALA and chitosan through deprotonated alginate that gave rise to the gathering of protoporphyrin IX (PpIX) for photodynamic detection within the cells. As per this research, chitosan-based nanoparticles in combination with alginate and folic acid are tremendous vectors for the definite delivery of 5-ALA to the CC cells to enable endoscopic fluorescent detection. Cathepsin B (CB) is strongly associated with the metastatic process and is available in surplus in the pericellular areas where this process occurs; thus, CB is important for the detection of metastasis. Ryu et al. [50] designed a CB-sensitive nanoprobe (CB-CNP) comprising a self-satisfied CB-CNP with a fluorogenic peptide attached to the tumor-targeting glycol chitosan nanoparticles (CNPs) on its surface. The designed nanoprobe is a sphere with a diameter of 280 nm, with spherical structure and its fluorescence capacity was completely extinguished under the biological condition. The evaluation of the usability of CB-sensitive nanoprobe in three rat metastatic models demonstrated the potential of these nonoprobes in discriminating metastatic cells from healthy ones through non-invasive imaging. Hyaluronic acid (HA) is another biopolymeric material. This is a biocompatible, negatively charged glycosaminoglycan, and is one of the main constituents of the extracellular matrix [51, 52]. HA can bind to the CD44 receptor, which is mostly over articulated in various cancerous cells, through the receptor-linker interaction. Thus, HA-modified nanoparticles are intriguing for their use in the detection and cure of cancer [53–55]. Wang et al. [56], coated the surface of iron oxide nanoparticles (IONP) with dopamine-modified HA. These nanoparticles have a hydrophilic exterior and a hydrophobic interior where the chemotherapeutic homocamptothecin is encapsulated [56]. The biopotential of this process was investigated in both laboratory and in the live cells. Increased uptake of nanoparticles by tumor cells was observed by MRI when an external magnetic field was employed [56]. After the intravenous administration of the nano-vehicle in 3 mg/kg (relative to the free drug) rats, a large tumor ablation was observed and after treatment, the tumors almost disappeared [56].

Choi et al. [53] also synthesized nanoparticles of hyaluronic acid with different diameters by changing the degree of hydrophobic replacement of HA. The nanoparticles were systemically administered in the mice with tumor, and then, its effect was studied. This same research group developed a versatile thermostatic system using poly (ethylene glycol) conjugated hyaluronic acid (P-HA-NPs) nanoparticles for the early detection of colon cancer and targeted therapy. To assess the effectiveness of the nanoparticles, they were first attached to the near-infrared fluorescent dye (Cy 5.5) by chemical conjugation, and then, the irinotecan anticancer drug (IRT) was encapsulated within these systems. The therapeutic potential of P-HA-NP was then investigated in different systems of the mice colon cancer. Through the intravenous injection of the fluorescent dye attached nanoparticles (Cy 5.5-P-HA-NPs), minute and initial-stage tumors as well as liver-embedded colon tumors were efficiently pictured using an NIRF imaging method. Due to their extraordinary capability to target tumors, drug-containing nanoparticles (IRT-P-HA-NP) showed markedly decreased tumor development with decreased systemic harmfulness. In addition, healing effects could be examined concurrently with Cy 5.5-P-HA-NPs [57].

Another option that can be used is alginate, which is a natural polymer derived from the brown seaweed and has been expansively scrutinized for its potential uses in the biomedical field because of its several favorable characteristics, such as low cost of manufacture, harmonious nature, less harmfulness, and easy gelling in response to the addition of divalent cations [58, 59]. Baghbani et al. [60] prepared perfluorohexane (PFH) nanodroplets stabilized with alginate to drive doxorubicin and then evaluated their sensitivity to ultrasound and imaging as well as their therapeutic properties. Further found that the ultrasound-facilitated treatment with PFH nanodroplets loaded with doxorubicin exhibited promising positive responses in the breast cancer rat models. The efficacy was characterized by the deterioration of the tumor [60]. In another study, Podgorna et al. [61] prepared gadolinium (GdNG) containing nanogels for hydrophilic drug loading and to enable screening by MRI. The gadolinium alginate nanogels had an average diameter of 110 nm with stability duration of 60 days. Because of their paramagnetic behavior, the gadolinium mixtures are normally used as positive contrast agents (T1) in the MRI images. Gadolinium nanogels significantly reduce the relaxation time (T1) compared to controls. Therefore, alginate nanogels act as contrast-enhancing agents and can be assumed as an appropriate material for pharmacological application.

Also, the polymeric material dextran is a neutral polymer and is assumed as the first notable example of microbial exopolysaccharides used in medical applications. A remarkable advantage of using dextran is that it is well-tolerated, non-toxic, and biodegradable in humans, with no reactions in the body [62]. Photodynamic therapy is a site-specific cancer cure with less damage to non-cancerous cells. Ding et al. [63] prepared a nanoparticulate multifunctional composite system by encapsulating Fe_3_O_4_ nanoparticles in dextran nanoparticles conjugated to redox-responsive chlorine 6 (C6) for near infrared (NIR) and magnetic resonance (MR) imaging. The nanoparticles exhibited an “off/on” behavior of the redox cellular response of the fluorescence signal, thus resulting in accurate imaging of the tumor. In addition, excellent in vitro and in vivo magnetic targeting ability was observed, contributing to the efficacy of enhanced photodynamic therapy. Hong et al. [64] prepared theranostic nanoparticles or glioma cells of C6 mice. These particles comprised of gadolinium oxide nanoparticles coated with folic acid-conjugated dextran (FA) or paclitaxel (PTX). The bioprotective effects of dextran coating and the chemotherapeutic effect of PTX on the C6 glioma cells were evaluated by the MTT assay. The synthesized nanoparticles have been shown to enter C6 tumor cells by receptor-mediated endocytosis and provide enhanced contrast (MR) concentration-dependent activity due to the paramagnetic property of the gadolinium nanoparticle. Multifunctional nanoparticles were more effective in reducing cell viability than uncoated gadolinium nanoparticles. Therefore, FA and PTX conjugated nanoparticles can be used as theranostic agents with paramagnetic and chemotherapeutic properties.

## Drug designing and drug delivery process and mechanism

With the progression of nanomedicine and, due to the advancement of drug discovery/design and drug delivery systems, numerous therapeutic procedures have been proposed and traditional clinical diagnostic methods have been studied, to increase the drug specificity and diagnostic accuracy. For instance, new routes of drug administration are being explored, and there is focus on ensuring their targeted action in specific regions, thus reducing their toxicity and increasing their bioavailability in the organism [65].

In this context, drug designing has been a promising feature that characterizes the discovery of novel lead drugs based on the knowledge of a biological target. The advancements in computer sciences, and the progression of experimental procedures for the categorization and purification of proteins, peptides, and biological targets are essential for the growth and development of this sector [66, 67]. In addition, several studies and reviews have been found in this area; they focus on the rational design of different molecules and show the importance of studying different mechanisms of drug release [68]. Moreover, natural products can provide feasible and interesting solutions to address the drug design challenges, and can serve as an inspiration for drug discovery with desired physicochemical properties [3, 69, 70].

Also, the drug delivery systems have been gaining importance in the last few years. Such systems can be easily developed and are capable of promoting the modified release of the active ingredients in the body. For example, Chen et al. [70] described an interesting review using nanocarriers for imaging and sensory applications and discussed the, therapy effect of these systems. In addition, Pelaz et al. [71] provided an up-to-date overview of several applications of nanocarriers to nanomedicine and discussed new opportunities and challenges for this sector.

**Fig. 2:**
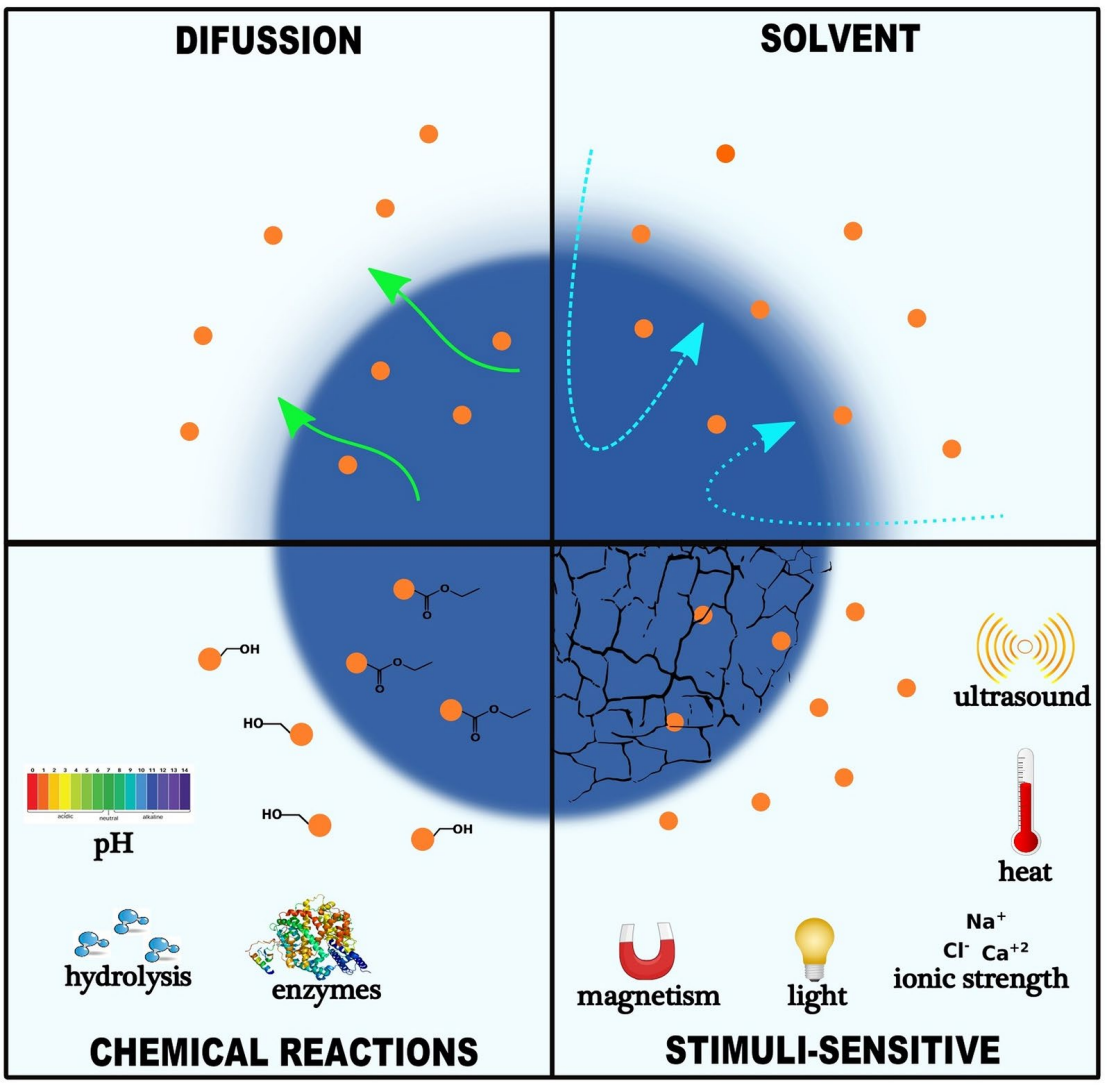
Mechanisms for controlled release of drugs using different types of nanocarriers

Interestingly, each of these drug delivery systems has its own chemical, physical and morphological characteristics, and may have affinity for different drugs polarities through chemical interactions (e.g., covalent bonds and hydrogen bonds) or physical interactions (e.g., electrostatic and van der Waals interactions). As an example, Mattos et al. [72] demonstrated that, the release profile of neem bark extract-grafted biogenic silica nanoparticles (chemical interactions) was lower than neem bark extract-loaded biogenic silica nanoparticles. Hence, all these factors influence the interaction of nanocarriers with biological systems [73], as well as the release kinetics of the active ingredient in the organism [68]. In addition, Sethi et al. [74] designed a crosslinkable lipid shell (CLS) containing docetaxel and wortmannin as the prototypical drugs used for controlling the drug discharge kinetics; then, they studied, its discharge profile, which was found to be affected in both in vivo and in vitro conditions. Apart from this, other parameters, such as the composition of the nanocarriers (e.g., organic, inorganic, and hybrid materials) and the form in which drugs are associated with them (such as core–shell system or matrix system) are also fundamental for understanding their drug delivery profile [75, 76]. Taken together, several studies regarding release mechanisms of drugs in nanocarriers have been conducted. Diffusion, solvent, chemical reaction, and stimuli-controlled release are a few mechanisms that can represent the release of drugs in nanocarriers as shown in Fig. 2 [77, 78]. Kamaly et al. [79] provided a widespread review of controlled-release systems with a focus on studies related to controlling drug release from polymeric nanocarriers.

Although there are several nanocarriers with different drug release profiles, strategies are currently being formulated to improve the specificity of the nanostructures to target regions of the organism [80], and to reduce the immunogenicity through their coating or chemical functionalization with several substances, such as polymers [81], natural polysaccharides [82, 83], antibodies [84], cell-membrane [85], and tunable surfactants [86], peptides [87], etc. In some cases where drugs do not display binding and affinity with a specific target or do not cross certain barriers (e.g. blood–brain barrier or the blood–cerebrospinal fluid barrier) [88], these ligand-modified nanocarriers have been used to pass through the cell membrane and allow a programmed drug delivery in a particular environment. For example, hyaluronic acid (a polysaccharide found in the extracellular matrix) has been used as a ligand-appended in several nanocarriers, showing promising results to boost antitumor action against the melanoma stem-like cells [89], breast cancer cells [90], pulmonary adenocarcinoma cells [91], as well as to facilitate intravitreal drug delivery for retinal gene therapy [83] and to reduce the immunogenicity of the formed protein corona [82]. However, the construction of the ligand-appended drug delivery systems is labor-intensive, and several targeting designs must be performed previously, taking into account the physiological variables of blood flow, disease status, and tissue architecture [92]. Moreover, few studies have been performed to evaluate the interaction of the ligand-appended in nanocarriers with cell membranes, and also their uptake mechanism is still unclear. Furthermore, has been known that the uptake of the nanoparticles by the cells occurs via phagocytic or non-phagocytic pathways (e.x. clathrin-mediated endocytosis, caveolae-mediated endocytosis, and others) [93, 94], meanwhile due some particular physicochemical characteristics of each delivery systems have been difficult to standardize the mechanism of action/interaction of these systems in the cells. For example, Salatin and Khosroushahi [95], in a review highlighted the main endocytosis mechanisms responsible for the cellular uptake of polysaccharide nanoparticles containing active compounds.

On the other hand, stimuli-responsive nanocarriers have shown the ability to control the release profile of drugs (as a triggered release) using external factors such as ultrasound [96], heat [97–99], magnetism [100, 101], light [102], pH [103], and ionic strength [104], which can improve the targeting and allow greater dosage control (Fig. 2). For example, superparamagnetic iron oxide nanoparticles are associated with polymeric nanocarriers [105] or lipids [106] to initially stimulate a controlled release system by the application of external magnetic field. In addition, Ulbrich et al. [107] revised recent achievements of drug delivery systems, in particular, on the basis of polymeric and magnetic nanoparticles, and also addressed the effect of covalently or noncovalently attached drugs for cancer cure [107]. Moreover, Au/Fe_3_O_4_@polymer nanoparticles have also been synthesized for the use in NIR-triggered chemo-photothermal therapy [108]. Therefore, hybrid nanocarriers are currently among the most promising tools for nanomedicine as they present a mixture of properties of different systems in a single system, thus ensuring materials with enhanced performance for both therapeutic and diagnostic applications (i.e., theranostic systems). Despite this, little is known about the real mechanisms of action and toxicity of drug delivery systems, which open opportunity for new studies. In addition, studies focusing on the synthesis of nanocarriers based on environmentally safe chemical reactions by implementing plant extracts and microorganisms have increased [10].

### Nanoparticles used in drug delivery system

#### Biopolymeric nanoparticles

There are numerous biopolymeric materials that are utilized in the drug delivery systems. These materials and their properties are discussed below.

##### Chitosan

Chitosan exhibits muco-adhesive properties and can be used to act in the tight epithelial junctions. Thus, chitosan-based nanomaterials are widely used for continued drug release systems for various types of epithelia, including buccal [109], intestinal [110], nasal [111], eye [112] and pulmonary [113]. Silva et al. [114] prepared and evaluated the efficacy of a 0.75% w/w isotonic solution of hydroxypropyl methylcellulose (HPMC) containing chitosan/sodium tripolyphosphate/hyaluronic acid nanoparticles to deliver the antibiotic ceftazidime to the eye. The rheological synergism parameter was calculated by calculating the viscosity of the nanoparticles in contact with mucin in different mass proportions. A minimum viscosity was observed when chitosan nanoparticles were placed in contact with mucin. However, the nanoparticles presented mucoadhesion which resulted in good interaction with the ocular mucosa and prolonged release of the antibiotic, and therefore, the nanoparticles can enhance the life span of the drug in the eyes. The nanoparticles did not show cytotoxicity for two cell lines tested (ARPE-19 and HEK 239T). The nanoparticles were also able to preserve the antibacterial activity, thus making them a promising formulations for the administration of ocular drugs with improved mucoadhesive properties.

Pistone et al. [115] prepared nanoparticles of chitosan, alginate and pectin as potential candidates for the administration of drugs into the oral cavity. The biocompatibility of the formulations was estimated based on the solubility of the nanoparticles in a salivary environment and its cytotoxicity potential was estimated in an oral cell line. Alginate nanoparticles were the most unwavering in the artificial saliva for at least 2 h, whereas pectin and especially chitosan nanoparticles were unstable. However, the chitosan nanoparticles were the most cyto-competitive, whereas alginate and pectin nanoparticles showed cytotoxicity under all tested conditions (concentration and time). The presence of Zn^2+^ (cross-linking agent) may be the cause of the observed cytotoxicity. Each formulation presented advantage and limitations for release into the oral cavity, thus necessitating their further refinement.

In addition, Liu et al. [116] prepared nanoparticles of carboxymethyl chitosan for the release of intra-nasal carbamazepine (CBZ) to bypass the blood–brain barrier membrane, thus increasing the amount of the medication in the brain and refining the treatment efficacy, thereby reducing the systemic drug exposure. The nanoparticles had a mean diameter of 218.76 ± 2.41 nm, encapsulation efficiency of 80% and drug loading of 35%. Concentrations of CBZ remained higher (P < 0.05) in the brain than the plasma over 240 min.

In another example, Jain and Jain [117] investigated the discharge profile of 5-fluorouracil (5-FU) from hyaluronic acid-coated chitosan nanoparticles into the gut, via oral administration. Release assays in conditions mimicking the transit from the stomach to the colon indicated the release profile of 5-FU which was protected against discharge in the stomach and small intestine. Also, the high local concentration of drugs would be able to increase the exposure time and thus, enhance the capacity for antitumor efficacy and decrease the systemic toxicity in the treatment of colon cancer.

##### Alginate

Another biopolymeric material that has been used as a drug delivery is alginate. This biopolymer presents final carboxyl groups, being classified as anionic mucoadhesive polymer and presents greater mucoadhesive strength when compared with cationic and neutral polymers [59, 118]. Patil and Devarajan [119] developed insulin-containing alginate nanoparticles with nicotinamide as a permeation agent in order to lower the serum glucose levels and raise serum insulin levels in diabetic rats. Nanoparticles administered sublingually (5 IU/kg) in the presence of nicotinamide showed high availability pharmacology (> 100%) and bioavailability (> 80%). The fact that NPs are promising carriers of insulin via the sublingual route have been proved in case of the streptozotocin-induced diabetic mouse model by achieving a pharmacological high potential of 20.2% and bio-availability of 24.1% compared to the subcutaneous injection at 1 IU/kg [119].

Also, Haque et al. [120] prepared alginate nanoparticles to release venlafaxine (VLF) via intranasal for treatment of depression. The higher blood/brain ratios of the VLF concentration to the alginate nanoparticles administered intra-nasally when compared to the intranasal VLF and VLF solution intravenously indicated the superiority of the nano-formulation in directly transporting the VLF to the brain. In this way, these nanoparticles are promising for the treatment of depression. In another example, Román et al. [121] prepared alginate microcapsules containing epidermal growth factor bound on its exterior part to target the non-small cell lung cancer cells. Cisplatin (carcinogen drug) was also loaded in the nanoparticles. The addition of EGF significantly increased specificity of carrier systems and presented kinetics of cell death (H460-lung cancer strain) faster than the free drug.

In addition, Garrait et al. [122] prepared nanoparticles of chitosan containing Amaranth red (AR) and subsequently microencapsulated these nanoparticles in alginate microparticles and studied the release kinetics of this new system in simulated gastric and intestinal fluids. The microparticles had a mean diameter of 285 μm with a homogeneous distribution; it was observed that there was a release of less than 5% of the AR contained in the systems in the gastric pH conditions, whereas the discharge was fast and comprehensive in the intestinal pH conditions. Thus, the carrier showed promise to protect molecules for intestinal release after oral administration.

Costa et al. [123] prepared chitosan-coated alginate nanoparticles to enhance the permeation of daptomycin into the ocular epithelium aiming for an antibacterial effect. In vitro permeability was assessed using ocular epithelial cell culture models. The antimicrobial activity of nanoencapsulated daptomycin showed potential over the pathogens engaged in bacterial endophthalmitis. Also, the ocular permeability studies demonstrated that with 4 h of treatment from 9 to 12% in total of daptomycin encapsulated in chitosan/alginate nanoparticles, these were able to cross the HCE and ARPE-19 cells. These results indicated that with this system an increasing in the drug retention in the ocular epithelium has occurred.

##### Xanthan gum

Xanthan gum (XG) is a high molecular weight heteropolysaccharide produced by *Xanthomonas campestris*. It is a polyanionic polysaccharide and has good bioadhesive properties. Because it is considered non-toxic and non-irritating, xanthan gum is widely used as a pharmaceutical excipient [124].

Laffleur and Michalek [125] have prepared a carrier composed of xanthan gum thiolated with l-cysteine to release tannin in the buccal mucosa to treat sialorrhea. Thiolation of xanthan gum resulted in increased adhesion on the buccal mucosa when compared to native xanthan gum. In addition, xanthan gum thiolate has a higher uptake of saliva whereas tannic acid ad-string and dry the oral mucosa. In this way, this system would be an efficient way of reducing the salivary flow of patients with sialorrhea. Angiogenesis is an important feature in regeneration of soft tissues.

Huang et al. [126] prepared injectable hydrogels composed of aldehyde-modified xanthan and carboxymethyl-modified chitosan containing potent angiogenic factor (antivascular endothelial growth factor, VEGF) to improve abdominal wall reconstruction. The hydrogel presented release properties mainly in tissues like digestive tract and open wounds. The hydrogel containing VEGF was able to accelerate the angiogenesis process and rebuild the abdominal wall. Menzel et al. [127] studied a new excipient aiming the use as nasal release system. Xanthan gum was used as a major polymer in which the-((2-amino-2-carboxyethyl) disulfanyl) nicotinic acid (Cys-MNA) was coupled. Characteristics, such as amount of the associated binder, mucoadhesive properties and stability against degradation, were analyzed in the resulting conjugate. Each gram of polymer was ligated with 252.52 ± 20.54 μmol of the binder. The muco-adhesion of the grafted polymer was 1.7 fold greater than that of thiolated xanthan and 2.5 fold greater than, that of native xanthan. In addition, the frequency of ciliary beating of nasal epithelial cells was poorly affected and was reversible only upon the removal of the polymer from the mucosa.

##### Cellulose

Cellulose and its derivatives are extensively utilized in the drug delivery systems basically for modification of the solubility and gelation of the drugs that resulted in the control of the release profile of the same [128]. Elseoud et al. [129] investigated the utilization of cellulose nanocrystals and chitosan nanoparticles for the oral releasing of repaglinide (an anti-hyperglycemic—RPG). The chitosan nanoparticles showed a mean size distribution of 197 nm while the hybrid nanoparticles of chitosan and cellulose nanocrystals containing RPG. Chitosan hybrid nanoparticles and oxidized cellulose nanocrystals containing RPG had a mean diameter of 251–310 nm. The presence of the hydrogen bonds between the cellulose nanocrystals and the drug, resulted in sustained release of the same, and subsequently the nanoparticles made with oxidized cellulose nanocrystals presented lower release when compared to the nanoparticles produced with native cellulose nanocrystals.

Agarwal et al. [130] have developed a drug targeting mechanism which is based on the conjugation of calcium alginate beads with carboxymethylcellulose (CMC) loaded 5-fluoroacyl (5-FU) and is targeted to the colon. The beads with lower CMC proportions presented greater swelling and muco-adhesiveness in the simulated colonic environment. With existence of colonic enzymes there was a 90% release of 5-FU encapsulated in the beads. Hansen et al. [131] investigated four cellulose derivatives, including, meteylcellulose, hydroxypropyl methylcellulose, sodium carboxymethylcellulose and cationic hydroxyethyl cellulose for application in drug release into the nasal mucosa. The association of these cellulose derivatives with an additional excipient, was also evaluated. The drug model employed in this process was acyclovir. The viability of the polymers as excipients for nasal release applications was also scrutinized for its ciliary beat frequency (CBF) and its infusion through the tissue system of the nostril cavity. An increase in thermally induced viscosity was observed when the cellulose derivatives were mixed with polymer graft copolymer. Further an increased permeation of acyclovir into the nasal mucosa was detected when it was combined with cationic hydroxyethylcellulose. None of the cellulose derivatives caused negative effects on tissues and cells of the nasal mucosa, as assessed by CBF.

##### Liposomes

They were discovered by Alec Bangham in 1960. Liposomes are used in the pharmaceutical and cosmetics industry for the transportation of diverse molecules and are among the most studied carrier system for drug delivery. Liposomes are an engrained formulation strategy to improve the drug delivery. They are vesicles of spherical form composed of phospholipids and steroids usually in the 50–450 nm size range [132]. These are considered as a better drug delivery vehicles since their membrane structure is analogous to the cell membranes and because they facilitate incorporation of drugs in them [132]. It has also been proved that they make therapeutic compounds stable, improve their biodistribution, can be used with hydrophilic and hydrophobic drugs and are also biocompatible and biodegradable. Liposomes are divided into four types: (1) conventional type liposomes: these consists of a lipid bilayer which can make either anionic, cationic, or neutral cholesterol and phospholipids, which surrounds an aqueous core material. In this case, both the lipid bilayer and the aqueous space can be filled with hydrophobic or hydrophilic materials, respectively. (2) PEGylated types: polyethylene glycol (PEG) is incorporated to the surface of liposome to achieve steric equilibrium, (3) ligand-targeted type: ligands like antibodies, carbohydrates and peptides, are linked to the surface of the liposome or to the end of previously attached PEG chains and (4) theranostic liposome type: it is an amalgamation kind of the previous three types of liposomes and generally consists of a nanoparticle along with a targeting, imaging and a therapeutic element [133].

The typical synthesis procedure for liposomes are as follows, thin layer hydration, mechanical agitation, solvent evaporation, solvent injection and the surfactant solubilization [134]. One aspect to point out on liposomes is that the drugs that are trapped within them are not bioavailable until they are released. Therefore, their accumulation in particular sites is very important to increase drug bioavailability within the therapeutic window at the right rates and times. Drug loading in liposomes is attained by active (drug encapsulated after liposome formation) and passive (drug encapsulated during liposome formation) approaches [135]. Hydrophilic drugs such as ampicillin and, 5-fluoro-deoxyuridine are typically confined in the aqueous core of the liposome and thus, their encapsulation does not depend on any modification in the drug/lipid ratio. However, the hydrophobic ones such as Amphotericin B, Indomethacin were found in the acyl hydrocarbon chain of the liposome and thus their engulfing are subjected to the characteristics of the acyl chain [136]. Among the passive loading approaches the mechanical and the solvent dispersion method as well as the detergent removal method can be mentioned [135].

There are obstacles with the use of liposomes for drug delivery purposes in the form of the RES (reticuloendothelial system), opsonization and immunogenicity although there are factors like enhanced permeability and EPR (retention effect) that can be utilized in order to boost the drug delivery efficiency of the liposomes [133, 135]. Once liposomes get into the body, they run into opsonins and high density lipoproteins (HDLs) and low density lipoproteins (LDLs) while circulating in the bloodstream by themselves. Opsonins (immunoglobulins and fibronectin, for example) assist RES on recognizing and eliminating liposomes. HDLs and LDLs have interactions with liposomes and decrease their stability. Liposomes tends to gather more in the sites like the liver and the spleen, this is an advantage because then a high concentration of liposomes can help treat pathogenic diseases, although in the case of cancers this can lead to a delay in the removal of lipophilic anticancer drugs. This is the reason why as mentioned at the beginning, different types of liposomes have been developed, in this case PEGylated ones. Dimov et al. [137] reported an incessant procedure of flow system for the synthesis, functionalization and cleansing of liposomes. This research consists of vesicles under 300 nm in a lab-on-chip that are useful and potential candidates for cost-intensive drugs or protein encapsulation development [137]. This is very important because costs of production also determine whether or not a specific drug can be commercialized. Liposome-based systems have now been permitted by the FDA [133, 135, 138–140].

##### Polymeric micelles

Polymeric micelles are nanostructures made of amphiphilic block copolymers that gather by itself to form a core shell structure in the aqueous solution. The hydrophobic core can be loaded with hydrophobic drugs (e.g. camptothecin, docetaxel, paclitaxel), at the same time the hydrophilic shell makes the whole system soluble in water and stabilizes the core. Polymeric micelles are under 100 nm in size and normally have a narrow distribution to avoid fast renal excretion, thus permitting their accumulation in tumor tissues through the EPR effect. In addition, their polymeric shell restrains nonspecific interactions with biological components. These nanostructures have a strong prospective for hydrophobic drug delivery since their interior core structure permits the assimilation of these kind of drugs resulting in enhancement of stability and bioavailability [141, 142].

Polymeric micelles are synthesized by two approaches: (1) convenient solvent-based direct dissolution of polymer followed by dialysis process or (2) precipitation of one block by adding a solvent [142, 143]. The factors like, hydrophobic chain size in the amphiphilic molecule, amphiphiles concentration, solvent system and temperature, affects the micelle formation [144]. The micelle assembly creation starts when minimum concentration known as the critical micelle concentration (CMC) is reached by the amphiphilic molecules [143]. At lower concentrations, the amphiphilic molecules are indeed small and occur independently [143]. Drugs are loaded within polymeric micelles by three common methodologies such as direct dissolution process, solvent evaporation process, and the dialysis process. As of the direct dissolution process, the copolymer and the drugs combine with each other by themselves in the water medium and forms a drug loaded with the micelles. While in the solvent evaporation process, the copolymer and the intended drug is dissolved using a volatile organic solvent and finally, in case of the dialysis process, both the drug in solution and the copolymer in the organic solvent are combined in the dialysis bag and then dialyzed with the formation of the micelle [145].

**Fig. 3:**
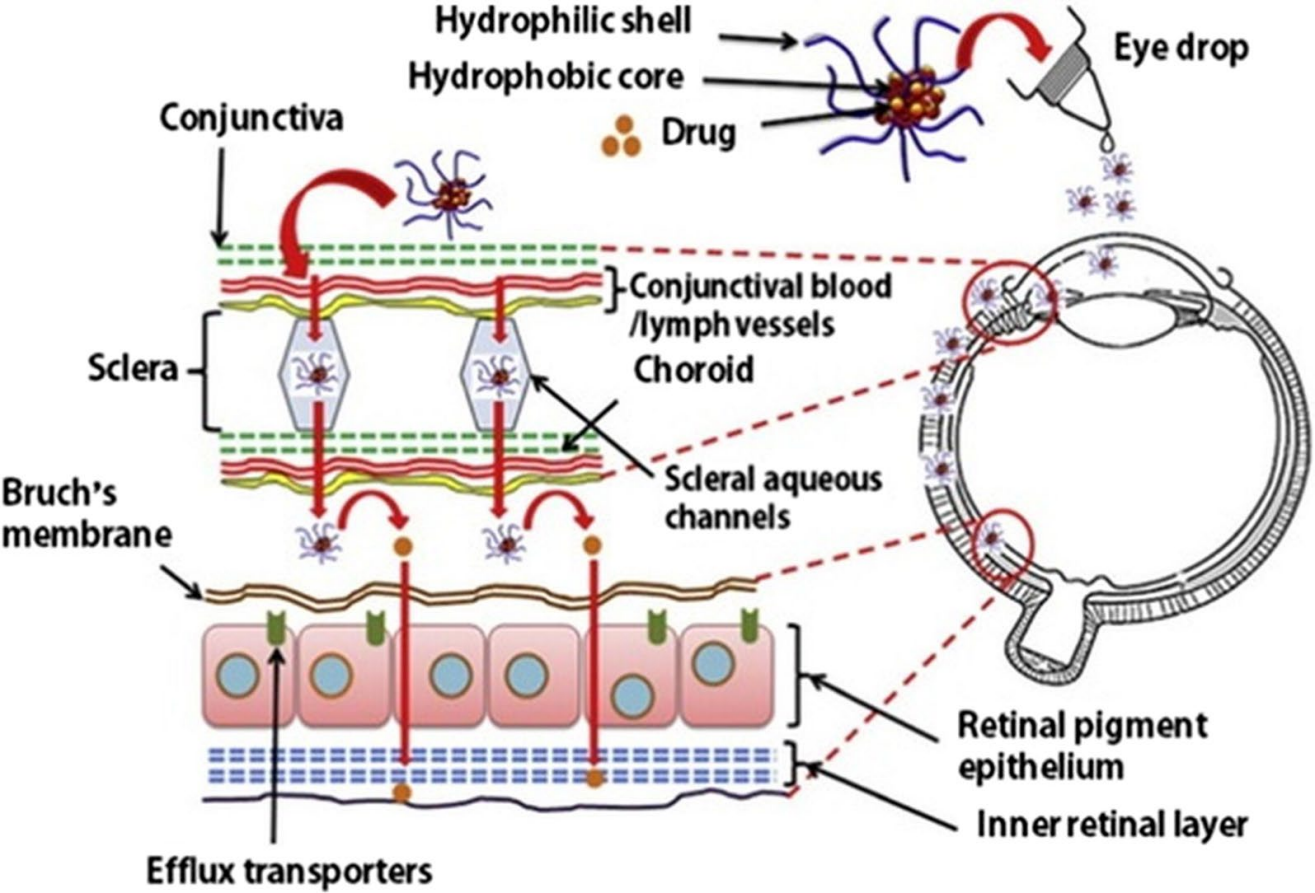
Polymeric micelles used for reaching the posterior ocular tissues via the transcleral pathway after topical application (the figure is reproduced from Mandal et al. [147] with required copyright permission)

The targeting of the drugs using different polymeric micelles as established by various mechanism of action including the boosted penetrability and the holding effect stimuli; complexing of a definite aiming ligand molecule to the surface of the micelle; or by combination of the monoclonal antibodies to the micelle corona [146]. Polymeric micelles are reported to be applicable for both drug delivery against cancer [143] and also for ocular drug delivery [147] as shown in Fig. 3 in which a polymeric micelle is used for reaching the posterior ocular tissues [147]. In the work by Li et al. [148], dasatinib was encapsulated within nanoparticles prepared from micellation of PEG-b-PC, to treat proliferative vitreoretinopathy (PVR), their size was 55 nm with a narrow distribution and they turned out to be noncytotoxic to ARPE-19 cells. This micellar formulation ominously repressed the cell proliferation, attachment and relocation in comparison to the free drugs [148]. The polymeric micelles is habitually get into the rear eye tissues through the transcleral pathway after relevant applications (Fig. 3; [147]).

##### Dendrimers

Dendrimers are highly bifurcated, monodisperse, well-defined and three-dimensional structures. They are globular-shaped and their surface is functionalized easily in a controlled way, which makes these structures excellent candidates as drug delivery agents [149–151]. Dendrimers can be synthesized by means of two approaches: The first one is the different route in which the dendrimer starts formation from its core and then it is extended outwards and the second is the convergent one, starts from the outside of the dendrimer [152]. Dendrimers are grouped into several kinds according to their functionalization moieties: PAMAM, PPI, liquid crystalline, core–shell, chiral, peptide, glycodendrimers and PAMAMOS, being PAMAM, the most studied for oral drug delivery because it is water soluble and it can pass through the epithelial tissue boosting their transfer via the paracellular pathway [153]. Dendrimers are limited in their clinical applications because of the presence of amine groups. These groups are positively charged or cationic which makes them toxic, hence dendrimers are usually modified in order to reduce this toxicity issue or to eliminate it. Drug loading in dendrimers is performed via the following mechanisms: Simple encapsulation, electrostatic interaction and covalent conjugation [154].

Drug is basically delivered by the dendrimers following two different paths, a) by the in vivo degradation of drug dendrimer’s covalent bonding on the basis of availability of suitable enzymes or favorable environment that could cleave the bonds and b) by discharge of the drug due to changes in the physical environment like pH, temperature etc., [154]. Dendrimers have been developed for transdermal, oral, ocular, pulmonary and in targeted drug delivery [155].

Jain et al. [156] have described the folate attached poly-l-lysine dendrimers (doxorubicin hydrochloride) as a capable cancer prevention drug carrier model for pH dependent drug discharge, target specificity, antiangiogenic and anticancer prospective, it was shown that doxorubicin-folate conjugated poly-l-lysine dendrimers increased the concentration of doxorubicin in the tumor by 121.5-fold after 24 h compared with free doxorubicin. Similarly, (Kaur et al. [157] developed folate-conjugated polypropylene imine dendrimers (FA-PPI) as a methotrexate (MTX) nanocarrier, for pH-sensitive drug release, selective targeting to cancer cells, and anticancer treatment. The in vitro studies on them showed sustained release, increased cell uptake and low cytotoxicity on MCF-7 cell lines [157]. Further, it has to be pointed out that the developed formulations, methotrexate (MTX)-loaded and folic acid-conjugated 5.0G PPI (MTX-FA-PPI), were selectively taken up by the tumor cells in comparison with the free drug, methotrexate (MTX).

##### Inorganic nanoparticles

Inorganic nanoparticles include silver, gold, iron oxide and silica nanoparticles are included. Studies focused on them are not as many as there are on other nanoparticle types discussed in this section although they show some potential applications. However, only few of the nanoparticles have been accepted for its clinical use, whereas the majority of them are still in the clinical trial stage. Metal nanoparticles, silver and gold, have particular properties like SPR (surface plasmon resonance), that liposomes, dendrimers, micelles do not possess. They showed several advantages such as good biocompatibility and versatility when it comes to surface functionalization.

Studies on their drug delivery-related activity have not been able to clear out whether the particulate or ionized form is actually related to their toxicity, and even though two mechanisms have been proposed, namely paracellular transport and transcytosis, there is not enough information about their in vivo transport and uptake mechanism [158]. Drugs can be conjugated to gold nanoparticles (AuNPs) surfaces via ionic or covalent bonding and physical absorption and they can deliver them and control their release through biological stimuli or light activation [159]. Silver nanoparticles exhibited antimicrobial activity, but as for drug delivery, very few studies have been carried out, for example, Prusty and Swain [160] synthesized an inter-linked and spongy polyacrylamide/dextran nano-hydrogels hybrid system with covalently attached silver nanoparticles for the release of ornidazole which turned out to have an in vitro release of 98.5% [160]. Similarly in another study, the iron oxide nanoparticles were synthesized using laser pyrolysis method and were covered with Violamycine B1, and antracyclinic antibiotics and tested against the MCF-7 cells for its cytotoxicity and the anti-proliferation properties along with its comparison with the commercially available iron oxide nanoparticles [161].

##### Nanocrystals

Nanocrystals are pure solid drug particles within 1000 nm range. These are 100% drug without any carriers molecule attached to it and are usually stabilized by using a polymeric steric stabilizers or surfactants. A nanocrystals suspension in a marginal liquid medium is normally alleviated by addition of a surfactant agent known as nano-suspension. In this case, the dispersing medium are mostly water or any aqueous or non-aqueous media including liquid polyethylene glycol and oils [162, 163]. Nanocrystals possesses specific characters that permit them to overcome difficulties like increase saturation solubility, increased dissolution velocity and increased glueyness to surface/cell membranes. The process by which nanocrystals are synthesized are divided into top-down and bottom-up approaches. The top-down approach includes, sono-crystallization, precipitation, high gravity controlled precipitation technology, multi-inlet vortex mixing techniques and limited impinging liquid jet precipitation technique [162]. However, use of an organic solvent and its removal at the end makes this process quite expensive. The bottom-up approach involves, grinding procedures along with homogenization at higher pressure [162]. Among all of the methods, milling, high pressure homogenization, and precipitation are the most used methods for the production of nanocrystals. The mechanisms by which nanocrystals support the absorption of a drug to the system includes, enhancement of solubility, suspension rate and capacity to hold intestinal wall firmly [162]. Ni et al. [164] embedded cinaciguat nanocrystals in chitosan microparticles for pulmonary drug delivery of the hydrophobic drug. The nanoparticles were contrived for continuous release of the drug taking advantage of the swelling and muco-adhesive potential of the polymer. They found that inhalation efficacy might be conceded under the disease conditions, so more studies are needed to prove that this system has more potential [164].

##### Metallic nanoparticles

In recent years, the interest of using metallic nanoparticles has been growing in different medical applications, such as bioimaging, biosensors, target/sustained drug delivery, hyperthermia and photoablation therapy [35, 165]. In addition, the modification and functionalization of these nanoparticles with specific functional groups allow them to bind to antibodies, drugs and other ligands, become these making these systems more promising in biomedical applications [166]. Although the most extensively studied, metallic nanoparticles are gold, silver, iron and copper, a crescent interest has been exploited regarding other kinds of metallic nanoparticles, such as, zinc oxide, titanium oxide, platinum, selenium, gadolinium, palladium, cerium dioxide among others [35, 165, 166].

##### Quantum dots

Quantum dots (QDs) are known as semiconductor nanocrystals with diameter range from 2 to 10 nm and their optical properties, such as absorbance and photoluminescence are size-dependent [167]. The QDs has gained great attention in the field of nanomedicine, since, unlike conventional organic dyes, the QDs presents emission in the near-infrared region (< 650 nm), a very desirable characteristic in the field of biomedical images, due to the low absorption by the tissues and reduction in the light scattering [167, 168]. In addition, QDs with different sizes and/or compositions can be excited by the same light source resulting in separate emission colors over a wide spectral range [169, 170]. In this sense, QDs are very appealing for multiplex imaging. In the medicine field QDs has been extensively studied as targeted drug delivery, sensors and bioimaging. A large number of studies regarding the applications of QDs as contrast agents for in vivo imaging is currently available in literature [168, 171–173]. Han et al. [172] developed a novel fluorophore for intravital cytometric imaging based on QDs-antibodies conjugates coated with norbornene-displaying polyimidazole ligands. This fluorophore was used to label bone marrow cells in vivo. The authors found that the fluorophore was able to diffuse in the entire bone marrow and label rare populations of cells, such as hematopoietic stem and progenitor cells [172]. Shi et al. [171] developed a multifunctional biocompatible graphene oxide quantum dot covered with luminescent magnetic nanoplatform for recognize/diagnostic of a specific liver cancer tumor cells (glypican-3-expressing Hep G2). According to the authors the attachment of an anti-GPC3-antibody to the nanoplataform results in selective separation of Hep G2 hepatocellular carcinoma cells from infected blood samples [171]. QDs could also bring benefits in the sustained and/or controlled release of therapeutic molecules. Regarding the controlled release, this behavior can be achieved via external stimulation by light, heat, radio frequency or magnetic fields [170, 174, 175]. Olerile et al. [176] have developed a theranostic system based on co-loaded of QDs and anti-cancer drug in nanostructured lipid carriers as a parenteral multifunctional system. The nanoparticles were spherical with higher encapsulation efficiency of paclitaxel (80.7 ± 2.11%) and tumor growth inhibition rate of 77.85%. The authors also found that the system was able to specifically target and detect H22 tumor cells [176]. Cai et al. [177] have synthesized pH responsive quantum dots based on ZnO quantum dots decorated with PEG and hyaluronic acid for become stable in physiological conditions and for targeting specific cells with HA-receptor CD44, respectively. This nanocarrier was also evaluated for doxorubicin (DOX) sustained release. The nanocarrier was stable in physiological pH and DOX was loaded in the carrier by forming complex with Zn^2+^ ions or conjugated to PEG. The DOX was released only in acidic intracellular conditions of tumor cells due to the disruption of ZnO QDs. The authors found that the anticancer activity was enhanced by the combination of DOX and ZnO QDs [177].

##### Protein and polysaccharides nanoparticles

**Fig. 4:**
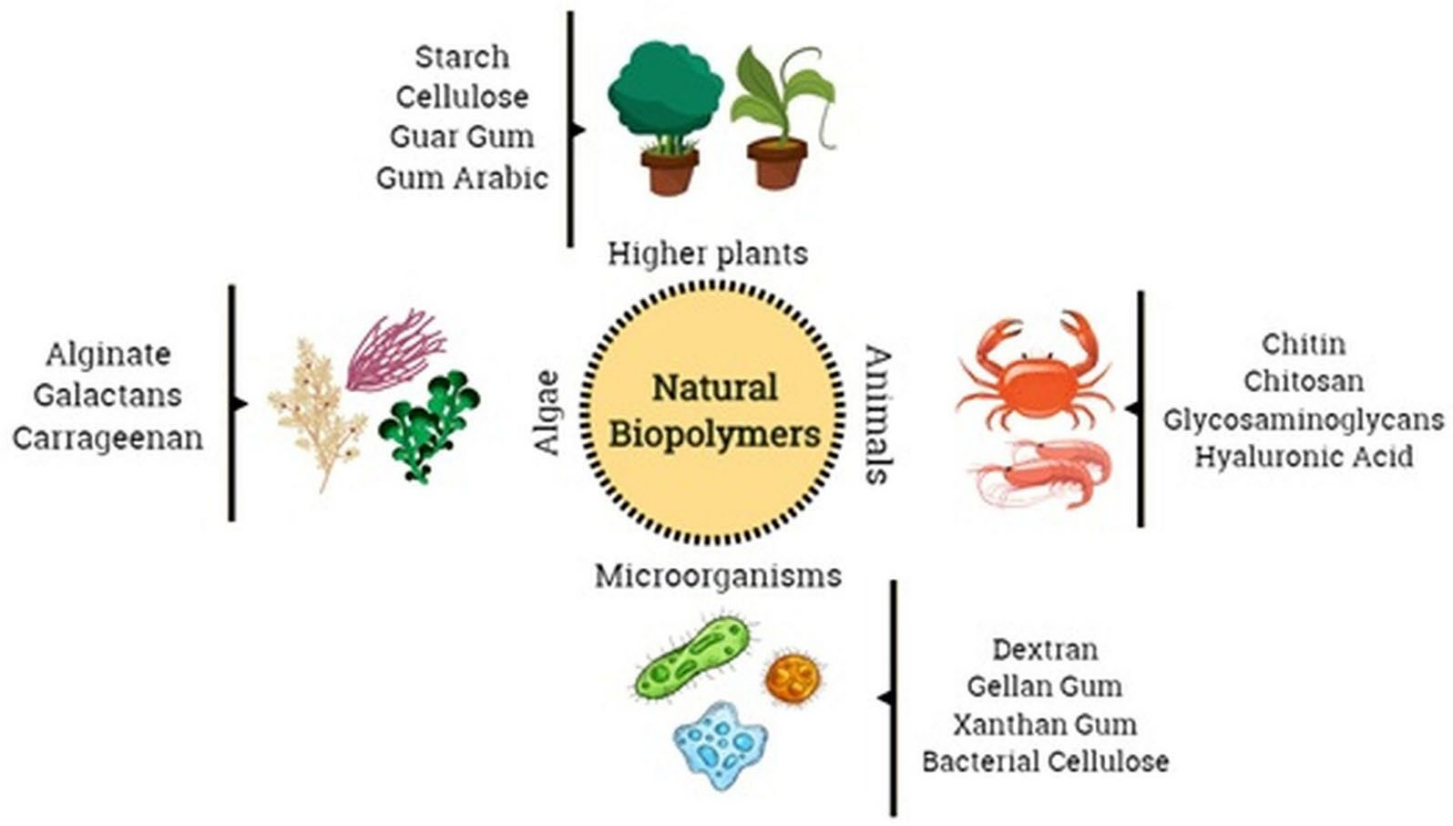
Different sources of natural biopolymers to be used in nanomedicine applications. Natural biopolymers could be obtained from higher plants, animals, microorganisms and algae

Polysaccharides and proteins are collectively called as natural biopolymers and are extracted from biological sources such as plants, animals, microorganisms and marine sources [178, 179]. Protein-based nanoparticles are generally decomposable, metabolizable, and are easy to functionalize for its attachment to specific drugs and other targeting ligands. They are normally produced by using two different systems, (a) from water-soluble proteins like bovine and human serum albumin and (b) from insoluble ones like zein and gliadin [180]. The usual methods to synthesize them are coacervation/desolvation, emulsion/solvent extraction, complex coacervation and electrospraying. The protein based nanoparticles are chemically altered in order to combine targeting ligands that identify exact cells and tissues to promote and augment their targeting mechanism [180]. Similarly, the polysaccharides are composed of sugar units (monosaccharides) linked through O-glycosidic bonds. The composition of these monomers as well as their biological source are able to confer to these polysaccharides, a series of specific physical–chemical properties [126, 179, 181]. One of the main drawback of the use of polysaccharides in the nanomedicine field is its degradation (oxidation) characteristics at high temperatures (above their melting point) which are often required in industrial processes. Besides, most of the polysaccharides are soluble in water, which limits their application in some fields of nanomedicine, such as tissue engineering [182, 183]. However, techniques such as crosslinking of the polymer chains have been employed in order to guarantee stability of the polysaccharide chains, guaranteeing them stability in aqueous environments [182, 183]. In Fig. 4, examples of some polysaccharides used in nanomedicine obtained from different sources are summarized. The success of these biopolymers in nanomedicine and drug delivery is due to their versatility and specified properties such as since they can originate from soft gels, flexible fibers and hard shapes, so they can be porous or non-porous; they have great similarity with components of the extracellular matrix, which may be able to avoid immunological reactions [179, 184].

There is not much literature related to these kind of nanoparticles, however, since they are generated from biocompatible compounds they are excellent candidates for their further development as drug delivery systems. Yu et al. [185] synthesized Bovine serum albumin and tested its attachment and/or infiltration property through the opening of the cochlea and middle ear of guinea pigs. The nanoparticles considered as the drug transporters were tested for their loading capacity and release behaviors that could provide better bio-suitability, drug loading capacity, and well-ordered discharge mechanism [185].

## Natural product-based nanotechnology and drug delivery

As per the World Health Organization (WHO) report, in developing countries, the basic health needs of approximately 80% of the population are met and/or complemented by traditional medicine [186]. Currently, the scientific community is focusing on the studies related to the bioactive compounds, its chemical composition and pharmacological potential of various plant species, to produce innovative active ingredients that present relatively minor side effects than existing molecules [5, 187]. Plants are documented as a huge sources of natural compounds of medicinal importance since long time and still it holds ample of resources for the discovery of new and highly effective drugs. However, the discovery of active compounds through natural sources is associated with several issues because they originate from living beings whose metabolite composition changes in the presence of stress. In this sense, the pharmaceutical industries have chosen to combine their efforts in the development of synthetic compounds [187–189]. Nevertheless, the number of synthetic molecules that are actually marketed are going on decreasing day by day and thus research on the natural product based active compounds are again coming to the limelight in spite of its hurdles [189, 190]. Most of the natural compounds of economic importance with medicinal potential that are already being marketed have been discovered in higher plants [187, 191]. Several drugs that also possess natural therapeutic agents in their composition are already available commercially; their applications and names are as follows: malaria treatment (Artemotil^®^ derived from *Artemisia annua* L., a traditional Chinese medicine plant), Alzheimer’s disease treatment (Reminyl^®^, an acetylcholinesterase inhibitor isolated from the *Galanthus woronowii* Losinsk), cancer treatment (Paclitaxel^®^ and its analogues derived from the *Taxus brevifolia* plant; vinblastine and vincristine extracted from *Catharanthus roseus*; camptothecin and its analogs derived from *Camptotheca acuminata* Decne), liver disease treatment (silymarin from *Silybum marianum*) [187].

The composition and activity of many natural compounds have already been studied and established. The alkaloids, flavonoids, tannins, terpenes, saponins, steroids, phenolic compounds, among others, are the bioactive molecules found in plants. However in most of the cases, these compounds have low absorption capacity due to the absence of the ability to cross the lipid membranes because of its high molecular sizes, and thus resulting in reduced bioavailability and efficacy [192]. These molecules also exhibit high systemic clearance, necessitating repeated applications and/or high doses, making the drug less effective for therapeutic use [189]. The scientific development of nanotechnology can revolutionize the development of formulations based on natural products, bringing tools capable of solving the problems mentioned above that limits the application of these compounds in large scale in the nanomedicine [7, 189]. Utilization of nanotechnology techniques in the medical field has been extensively studied in the last few years [193, 194]. Hence these can overcome these barriers and allow different compounds and mixtures to be used in the preparation of the same formulation. In addition, they can change the properties and behavior of a compound within the biological system [7, 189]. Besides, bringing benefits to the compound relative to the solubility and stability of the compounds, release systems direct the compound to the specific site, increase bioavailability and extend compound action, and combine molecules with varying degrees of hydrophilicity/lipophilicity [7]. Also, there is evidence that the association of release systems with natural compounds may help to delay the development of drug resistance and therefore plays an important role in order to find new possibilities for the treatment of several diseases that have low response to treatment conventional approaches to modern medicine [7, 189].

**Fig. 5:**
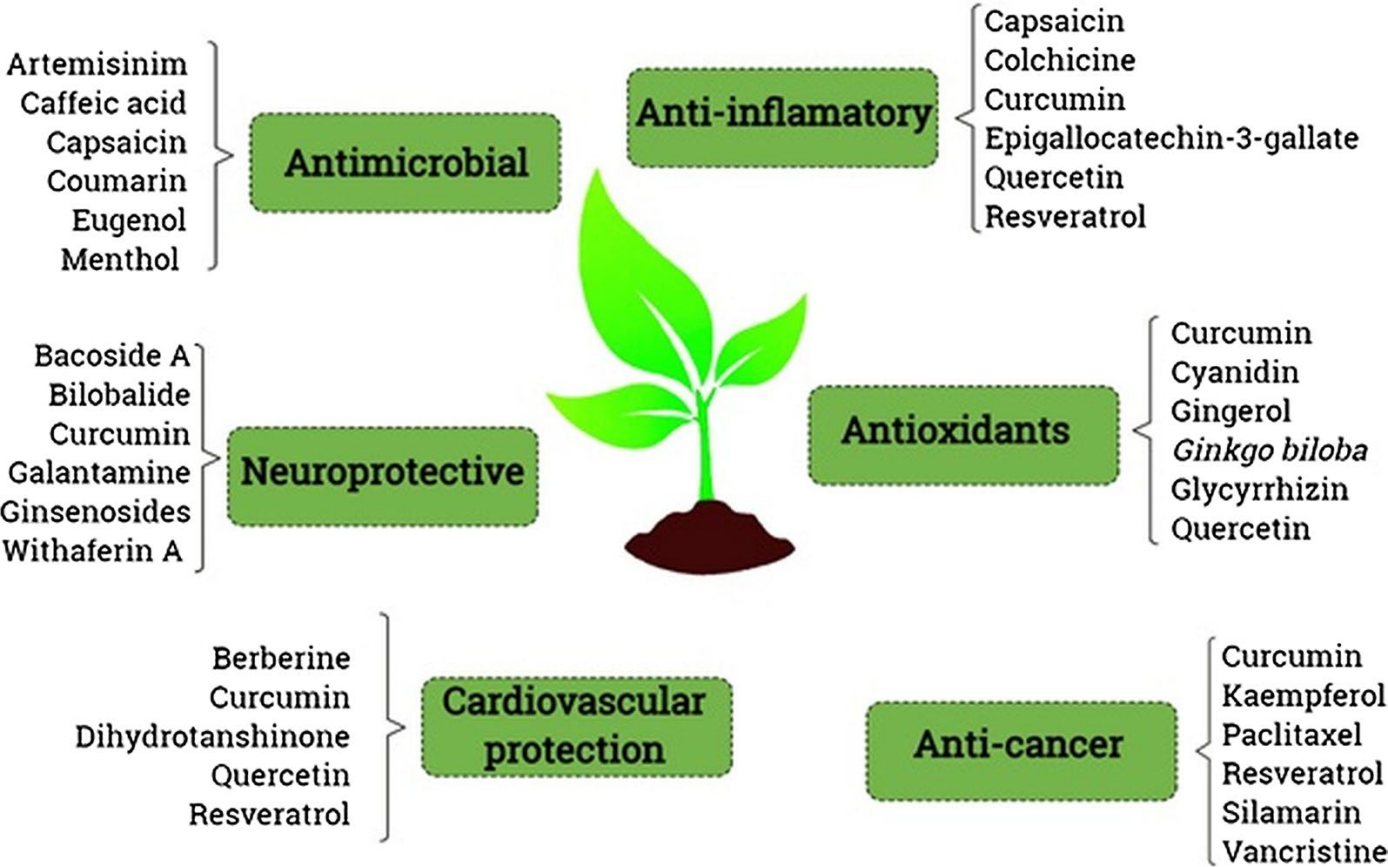
Examples of natural compounds extracted from higher plants used in nanomedicine aiming different approaches. Some of these extracts are already being marketed, others are in clinical trials and others are being extensively studied by the scientific community

The natural product based materials are of two categories, (1) which are targeted to specific location and released in the specific sites to treat a number of diseases [43, 195] and (2) which are mostly utilized in the synthesis process [196]. Most of the research is intended for treatment against the cancer disease, since it is the foremost reason of death worldwide nowadays [197, 198]. In case of the cancer disease, different organs of the body are affected, and therefore the need for the development of an alternative medicine to target the cancerous cells is the utmost priority among the modern researchers, however, a number of applications of nanomedicine to other ailments is also being worked on [199, 200]. These delivery systems are categorized in terms of their surface charge, particle size, size dispersion, shape, stability, encapsulation potential and biological action which are further utilized as per their requirements [33]. Some examples of biological compounds obtained from higher plants and their uses in the nanomedicine field are described in Fig. 5. Pharmaceutical industries have continuously sought the development and application of new technologies for the advancement and design of modern drugs, as well as the enhancement of existing ones [71, 201]. In this sense, the accelerated development of nanotechnology has driven the design of new formulations through different approaches, such as, driving the drug to the site of action (nanopharmaceutics); image and diagnosis (nanodiagnostic), medical implants (nanobiomaterials) and the combination diagnosis and treatment of diseases (nanotheranostics) [71, 202, 203].

Currently, many of the nanomedicines under development, are modified release systems for active ingredients (AI) that are already employed in the treatment of patients [203, 204]. For this type of approach, it is evaluated whether the sustained release of these AIs modifies the pharmacokinetic profile and biodistribution. In this context, it can be ascertained that the nano-formulation offers advantages over the existing formulation if the AI is directed towards the target tissue shows increased uptake/absorption by the cells and lower toxicity profile for the organism [205, 206]. This section is focused on berberine, curcumin, ellagic acid, resveratrol, curcumin and quercetin [8]. Some other compounds mentioned are doxorubicin, paclitaxel and vancomycin that also come from natural products.

Nanoparticles have been synthesized using natural products. For example, metallic, metal oxide and sulfides nanoparticles have been reported to be synthesized using various microorganisms including bacteria, fungi, algae, yeast and so on [207] or plant extracts [208]. For the first approach, the microorganism that aids the synthesis procedure is prepared in the adequate growth medium and then mixed with a metal precursor in solution and left for incubation to form the nanoparticles either intracellularly or extracellularly [209–211]. As for the second approach, the plant extract is prepared and mixed afterwards with the metal precursor in solution and incubated further at room temperature or boiling temperature for a definite time or exposed to light as an external stimulus to initiate the synthesis of nanoparticles [212].

Presently, these natural product based materials are considered as the key ingredients in the preparation and processing of new nano-formulations because they have interesting characteristics, such as being biodegradable, biocompatible, availability, being renewable and presenting low toxicity [178, 179, 213]. In addition to the aforementioned properties, biomaterials are, for the most part, capable of undergoing chemical modifications, guaranteeing them unique and desirable properties for is potential uses in the field of nanomedicine [45, 214]. Gold, silver, cadmium sulfide and titanium dioxide of different morphological characteristics have been synthesized using a number of bacteria namely *Escherichia coli*, *Pseudomonas aeruginosa*, *Bacillus subtilis* and *Klebsiella pneumoniae* [211]. These nanoparticles, especially the silver nanoparticles have been abundantly studied in vitro for their antibacterial, antifungal, and cytotoxicity potential due to their higher potential among all metal nanoparticles [215, 216]. In the event of microorganism mediated nanoparticle synthesis, maximum research is focused on the way that microorganisms reduce metal precursors and generate the nanoparticles. For instance, Rahimi et al. [217] synthesized silver nanoparticles using *Candida albicans* and studied their antibacterial activity against two pathogenic bacteria namely *Staphylococcus aureus* and *E. coli.* Similarly, Ali et al. [218] synthesized silver nanoparticles with the *Artemisia absinthium* aqueous extract and their antimicrobial activity was assessed versus *Phytophthora parasitica* and *Phytophthora capsici* [218]. Further, Malapermal et al. [219] used *Ocimum basilicum* and *Ocimum sanctum* extracts to synthesize nanoparticles and studied its antimicrobial potential against *E. coli*, *Salmonella* spp., *S. aureus*, and *P. aeruginosa* along with the antidiabetic potential. Likewise, Sankar et al. [220] also tested the effect of silver nanoparticles for both antibacterial and anticancer potential against human lung cancer cell line. Besides the use of microorganism, our group has synthesized silver, gold and iron oxide nanoparticles using various food waste materials such as extracts of *Zea mays* leaves [221, 222], onion peel extract [223], silky hairs of *Zea mays* [224], outer peel of fruit of *Cucumis melo* and *Prunus persica* [225], outer peel of *Prunus persica* [226] and the rind extract of watermelon [227], etc. and have tested their potential antibacterial effects against various foodborne pathogenic bacteria, anticandidal activity against a number of pathogenic *Candida* spp., for their potential antioxidant activity and proteasome inhibitory effects.

**Fig. 6:**
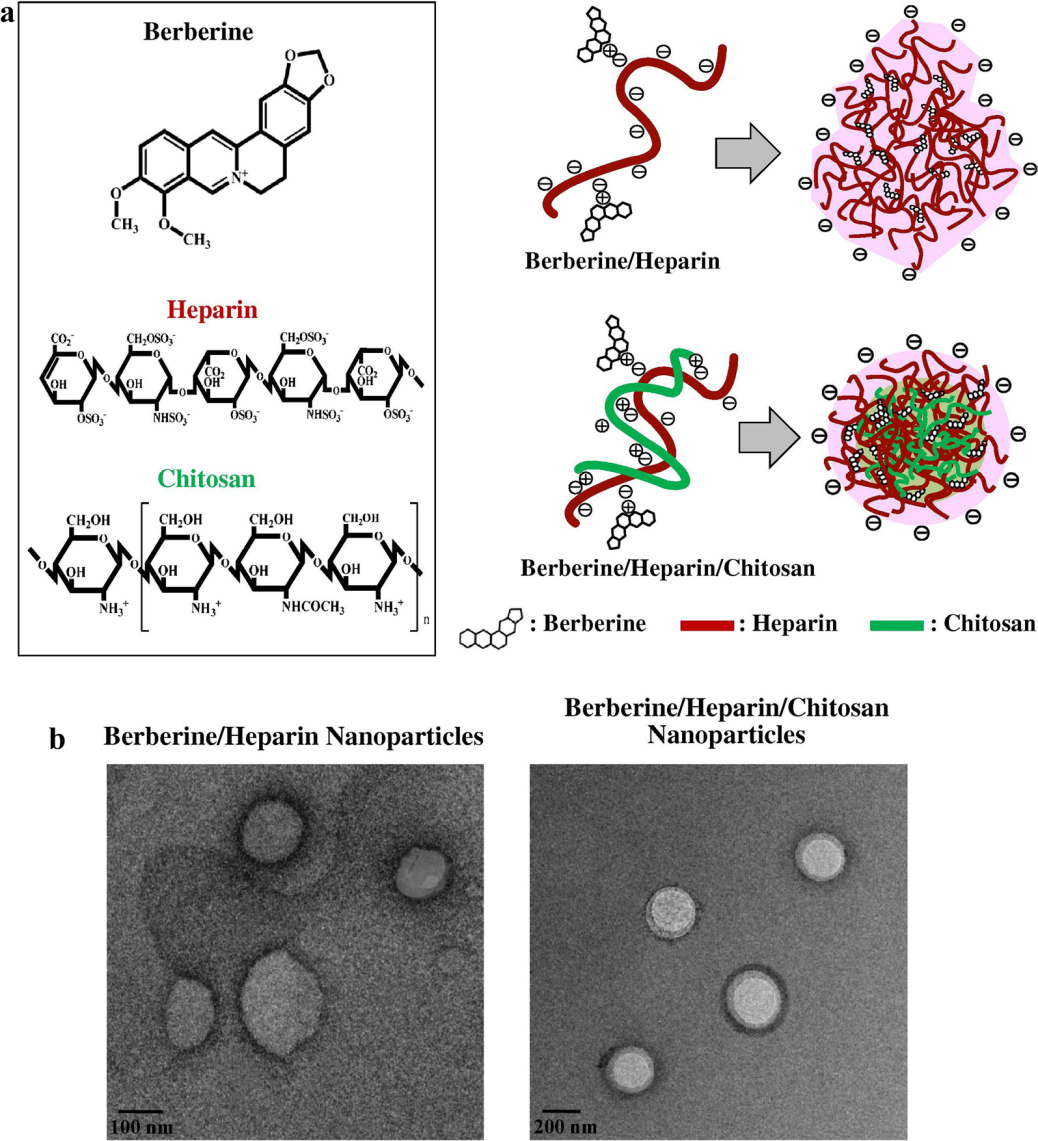
**a** Structure of berberine/heparin based nanoparticles and berberine/heparin/chitosan nanoparticles. **b** TEM images of the berberine/heparin nanoparticles and berberine/heparin/chitosan nanoparticles (the figure is reproduced from Chang et al. [232] with required copyright permission)

For drug delivery purposes, the most commonly studied nanocarriers are crystal nanoparticles, liposomes, micelles, polymeric nanoparticles, solid lipid nanoparticles, superparamagnetic iron oxide nanoparticles and dendrimers [228–230]. All of these nanocarriers are formulated for natural product based drug delivery. For applications in cancer treatment, Gupta et al. [231] synthesized chitosan based nanoparticles loaded with Paclitaxel (Taxol) derived from *Taxus brevifolia*, and utilized them for treatment of different kinds of cancer. The authors concluded that the nanoparticle loaded drug exhibited better activity with sustained release, high cell uptake and reduced hemolytic toxicity compared with pure Paclitaxel [231]. Berberine is an alkaloid from the barberry plant. Chang et al. [232] created a heparin/berberine conjugate to increase the suppressive *Helicobacter pylori* growth and at the same time to reduce cytotoxic effects in infected cells [232] which is depicted in Fig. 6.

Aldawsari and Hosny [233] synthesized ellagic acid-SLNs to encapsulate Vancomycin (a glycopeptide antibiotic produced in the cultures of *Amycolatopsis orientalis*). Further, its in vivo tests were performed on rabbits and the results indicated that the ellagic acid prevented the formation of free oxygen radicals and their clearance radicals, thus preventing damages and promoting repair [233]. Quercetin is a polyphenol that belongs to the flavonoid group, it can be found in citrus fruits and vegetables and it has antioxidant properties. In a study by Dian et al. [234], polymeric micelles was used to deliver quercetin and the results showed that such micelles could provide continuous release for up to 10 days in vitro, with continuous plasma level and boosted complete accessibility of the drug under in vivo condition [234].

Daunorubicin is a natural product derived from a number of different wild type strains of *Streptomyces*, doxorubicin (DOX) is a hydrolated version of it used in chemotherapy [213]. Spillmann et al. [235] developed a multifunctional liquid crystal nanoparticle system for intracellular fluorescent imaging and for the delivery of doxorubicin in which the nanoparticles were functionalized with transferrin. Cellular uptake and sustained released were attained within endocytic vesicles in HEK 293T/17 cells. Perylene was used as a chromophore to track the particles and to encapsulate agents aimed for intracellular delivery [235]. Purama et al. [236] extracted dextran from two sucrose based lactic acid bacteria namely *Streptococcus mutans* and *Leuconostoc mesenteroides*. Agarwal et al. [237] formulated a dextran-based dendrimer formulation and evaluated its drug discharge capacity and haemolytic activity under in vitro condition. They concluded that the dendritic structure selectively enters the highly permeable portion of the affected cells without disturbing the healthy tissues thereby making more convenient for its application in the biomedical field [237]. Folate- functionalized superparamagnetic iron oxide nanoparticles developed previously for liver cancer cure are also been used for the delivery of Doxil (a form of doxorubicin which was the first FDA-approved nano-drug in 1995) [238]. The in vivo studies in rabbits and rats showed a two- and fourfold decrease compared with Doxil alone while folate aided and enhanced specific targeting [239]. Liposomes are the nanostructures that have been studied the most, and they have been used in several formulations for the delivery of natural products like resveratrol [240]. Curcumin, a polyphenolic compound obtained from turmeric, have been reported to be utilized in the cure of cancers including the breast, bone, cervices, liver, lung, and prostate [241]. Liposomal curcumin formulations have been developed for the treatment of cancer [242, 243]. Cheng et al. [244] encapsulated curcumin in liposomes by different methods and compared the outcomes resulting that the one dependent on pH yielded stable products with good encapsulation efficiency and bio-accessibility with potential applications in cancer treatment [244].

Over all, it can be said that the sustained release systems of naturally occurring therapeutic compounds present themselves as a key tools for improving the biological activity of these compounds as well as minimizing their limitations by providing new alternatives for the cure of chronic and terminal diseases [8, 245]. According to BBC Research, the global market for plant-derived pharmaceuticals will increase from $29.4 billion in 2017 to about $39.6 billion in 2022 with a compound annual growth rate (CAGR) of 6.15% in this period (BCC-RESEARCH). Some of nanostructure-based materials covered in this section have already been approved by the FDA. Bobo et al. [255] has provided the information on nanotechnology-based products already approved by the FDA (Table 1).

**Table 1:** List of FDA-approved nanotechnology-based products and clinical trials. The table is reproduced from Bobo et al. [255] with required copyright permission

Polymer nanoparticles-synthetic polymer particles combined with drugs or biologics				
Name	Material description	Nanoparticle advantage	Indication(s)	Year approved
Adagen^®^/pegademase bovine (Sigma-Tau Pharmaceuticals)	PEGylated adenosine deaminase enzyme	Improve circulation time and decreased immunogenicity	Severe combined immunodeficiency disease (SCID)	1990
Cimzia^®^/certolizumab pegol (UCB)	PEGylated antibody fragment (Certolizumab)	Improved circulation time and greater stability in vivo	Crohn’s disease; Rheumatoid arthritis; Psoriatic Arthritis; Ankylosing Spondylitis	2008; 2009; 2013; 2013
Copaxone^®^/Glatopa (Teva)	Random copolymer of l-glutamate, l alanine, l-lysine and l-tyrosine	Large amino-acid based polymer with controlled molecular weight and clearance characteristics	Multiple sclerosis (MS)	1996
Eligard^®^ (Tolmar)	Leuprolide acetate and polymer (PLGH (poly (dl-lactide-coglycolide))	Controlled delivery of payload with longer circulation time	Prostate cancer	2002
Macugen^®^/Pegaptanib (Bausch & Lomb)	PEGylated anti-VEGF aptamer (vascular endothelial growth factor) aptamer	Improved stability of aptamer as a result of PEGylation	Macular degeneration, neovascular age-related	2004
Mircera^®^/Methoxy polyethylene glycol-epoetin beta (Hoffman-La Roche)	Chemically synthesized ESA (erythropoiesis-stimulating agent)	Improved stability of aptamer as a result of PEGylation	Anemia associated with chronic kidney disease	2007
Neulasta^®^/pegfilgrastim (Amgen)	PEGylated GCSF protein	Improved stability of protein through PEGylation	Neutropenia, chemotherapy induced	2002
Pegasys^®^ (Genentech)	PEGylated IFN alpha-2a protein	Improved stability of protein through PEGylation	Hepatitis B; Hepatitis C	2002
PegIntron^®^ (Merck)	PEGylated IFN alpha-2a protein	Improved stability of protein through PEGylation	Hepatitis C	2001
Renagel^®^[sevelamer hydrochloride]/Renagel^®^[sevelamer carbonate] (Sanofi)	Poly(allylamine hydrochloride)	Increase circulation and therapeutic delivery	Chronic kidney disease	2000
Somavert^®^/pegvisomant (Pfizer)	PEGylated HGH receptor antagonist	Improved stability of protein through PEGylation	Acromegaly	2003
Oncaspar^®^/pegaspargase (Enzon pharmaceuticals)	Polymer–protein conjugate (PEGylated l-asparaginase)	Improved stability of protein through PEGylation	Acute lymphoblastic leukemia	1994
Krystexxa^®^/pegloticase (Horizon)	Polymer–protein conjugate (PEGylated porcine-like uricase)	Improved stability of protein through PEGylation; introduction of unique mammalian protein	Chronic gout	2010
Plegridy^®^ (Biogen)	Polymer–protein conjugate (PEGylated IFN beta-1a)	Improved stability of protein through PEGylation	Multiple Sclerosis	2014
ADYNOVATE (Baxalta)	Polymer–protein conjugate (PEGylated factor VIII)	Improved stability of protein through PEGylation	Hemophilia	2015
Zilretta	Triamcinolone acetonide with a poly lactic-*co*-glycolic acid (PLGA) matrix microspheres	Extended pain relief over 12 weeks	Osteoarthritis (OA) of the knee	2017
Rebinyn	Coagulation fator IX (Recombinant) GlycoPEGylated	Effective control in 95% of bleeding episodes; 98% of bleeds were treated with 1–2 infusions	Control and prevention of bleeding episodes and prevention of bleeding in the perioperative setting for haemophilia B patients	2017
Liposome formulations combined with drugs or biologics				
DaunoXome^®^ (Galen)	Liposomal daunorubicin	Increased delivery to tumour site; lower systemic toxicity arising from side-effects	Karposi’s sarcoma	1995
DepoCyt© (Sigma-Tau)	Liposomal cytarabine	Increased delivery to tumour site; lower systemic toxicity arising from side-effects	Lymphomatous meningitis	1996
Marqibo^®^ (Onco TCS)	Liposomal vincristine	Increased delivery to tumour site; lower systemic toxicity arising from side-effects	Acute lymphoblastic leukemia	2012
Onivyde^®^ (Merrimack)	Liposomal irinotecan	Increased delivery to tumour site; lower systemic toxicity arising from side-effects	Pancreatic cancer	2015
AmBisome^®^ (Gilead Sciences)	Liposomal amphotericin B	Reduced nephrotoxicity	Fungal/protozoal infections	1997
DepoDur^®^ (Pacira Pharmaceuticals)	Liposomal morphine sulphate	Extended release	Analgesia (post-operative)	2004
Visudyne^®^ (Bausch and Lomb)	Liposomal verteporfin	Increased delivery to site of diseased vessels; photosensitive release	Macular degeneration, wet age-related; myopia; ocular histoplasmosis	2000
Doxil^®^/Caelyx™ (Janssen)	Liposomal doxorubicin	Improved delivery to site of disease; decrease in systemic toxicity of free drug	Karposi’s sarcoma; Ovarian cancer; multiple myeloma	1995; 2005; 2008
Abelcet^®^ (Sigma-tau)	Liposomal amphotericin B lipid complex	Reduced toxicity	Fungal infections	1995
Curosurf^®^/Poractant alpha (Chiesei farmaceutic	Liposome–proteins SP-B and SP-C	Increased delivery for smaller volume; reduced toxicity	Pulmonary surfactant for respiratory distress syndrome	1999
Vyxeos (Jazz Pharma)	Lipossomal combination of daunorubicin and cytarabine	Sustained release of the molecules and co-loaded two molecules with synergistic anti-tumor activity	Acute myeloid leukemia (AML) or AMLA with myelodysplasia-related changes (AML-MRC)	2017
Micellar nanoparticles combined with drugs or biologics				
Estrasorb™ (Novavax)	Micellar estradiol	Controlled delivery of therapeutic	Menopausal therapy	2003
Protein nanoparticles combined with drugs or biologics				
Abraxane^®^/ABI-007 (Celgene)	Albumin-bound paclitaxel nanoparticles	Improved solubility; improved delivery to tumor	Breast cancer; NSCLC; Pancreatic cancer	2005; 2012; 2013
Ontak^®^ (Eisai Inc)	Engineered protein combining IL-2 and diphtheria toxin	Targeted T cell specificity; lysosomal escape	Cutaneous T-cell lymphoma	1999
Nanocrystals				
Emend^®^ (Merck)	Aprepitant	Surface area allows faster absorption and increases bioavailability	Antiemetic	2003
Tricor^®^ (Lupin Atlantis)	Fenofibrate	Increases bioavailability simplifies administration	Hyperlipidemia	2004
Rapamune^®^ (Wyeth Pharmaceuticals)	Sirolimus	Increased bioavailability	Immunosuppressant	2000
Megace ES^®^ (Par Pharmaceuticals)	Megestrol acetate	Reduced dosing	Anti-anorexic	2001
Avinza^®^ (Pfizer)	Morphine sulphate	Increased drug loading and bioavailability; extended release	Psychostimulant	2002 (2015)
Focalin XR^®^ (Novartis)	Dexmethylphenidate HCl	Increased drug loading and bioavailability	Psychostimulant	2005
Ritalin LA^®^ (Novartis)	Methylphenidate HCl	Increased drug loading and bioavailability	Psychostimulant	2002
Zanaflex^®^ (Acorda)	Tizanidine HCl	Increased drug loading and bioavailability	Muscle relaxant	2002
Vitoss^®^ (Stryker)	Calcium phosphate	Mimics bone structure allowing cell adhesion and growth	Bone substitute	2003
Ostim^®^ (Heraseus Kulzer)	Hydroxyapatite	Mimics bone structure allowing cell adhesion and growth	Bone substitute	2004
OsSatura^®^ (IsoTis Orthobiologics)	Hydroxyapatite	Mimics bone structure allowing cell adhesion and growth	Bone substitute	2003
NanOss^®^ (Rti surgical)	Hydroxyapatite	Mimics bone structure allowing cell adhesion and growth	Bone substitute	2005
EquivaBone^®^ (Zimmer Biomet)	Hydroxyapatite	Mimics bone structure	Bone substitute	2009
Invega^®^ Sustenna^®^ (Janssen Pharms)	Paliperidone palmitate	Allows slow release of injectable low solubility drug	Schizophrenia; Schizoaffective disorder	2009; 2014
Ryanodex^®^ (Eagle Pharmaceuticals)	Dantrolene sodium	Faster administration at higher doses	Malignant hypothermia	2014
Inorganic and metallic nanoparticles Nanotherm^®^ (MagForce)	Iron oxide	Allows cell uptake and introduces superparamagnetism	Glioblastoma	2010
Feraheme™/ferumoxytol (AMAG pharmaceuticals)	Ferumoxytol SPION with polyglucose sorbitol carboxymethylether	Magnetite suspension allows for prolonged steady release, decreasing number of doses	Deficiency anemia iron deficiency in chronic kidney disease (CKD)	2009
Venofer^®^ (Luitpold pharmaceuticals)	Iron sucrose	Allows increased dose	Iron deficiency in chronic kidney disease (CKD)	2000
Ferrlecit^®^ (Sanofi Avertis)	Sodium ferric gluconate	Allows increased dose	Iron deficiency in chronic kidney disease (CKD)	1999
INFeD^®^ (Sanofi Avertis)	Iron dextran (low MW)	Allows increased dose	Iron deficiency in chronic kidney disease (CKD)	1957
DexIron^®^/Dexferrum^®^ (Sanofi Avertis)	Iron dextran (low MW)	Allows increased dose	Iron deficiency in chronic kidney disease (CKD)	1957
Feridex^®^/Endorem^®^ (AMAG pharmaceuticals)	SPION coated with dextran	Superparamagnetic character	Imaging agent	1996 (2008)
GastroMARK™; umirem^®^ (AMAG pharmaceuticals)	SPION coated with silicone	Superparamagnetic character	Imaging agent	2001 (2009)

Data from 2016–2018 has been collected from various literature [204, 256–261]

## Regulation and reality: products now on the market

In the current medical nanotechnology scenario, there are 51 products based on this technology [204, 246–248] which are currently being applied in clinical practice (Table 2). Notably, such nanomedicines are primarily developed for drugs, which have low aqueous solubility and high toxicity, and these nanoformulations are often capable of reducing the toxicity while increasing the pharmacokinetic properties of the drug in question.

**Table 2:** Nanomedicine approved by FDA classified by type of carrier/material used in preparation of the formulation

Commercial name (company)	Ingredient active	Carrier	Application	Advantage	Year approved
Doxil^®^/Caelyx™ (Janssen)	Doxorubicin	Liposomes	Karposi’s sarcoma; Ovarian cancer; multiple myeloma	Increase site-specific delivery (tumor) and decrease systemic toxicity	1995; 2005; 2008
Abelcet^®^ (Sigma-tau)	Amphotericin B lipid complex	Liposomes	Fungal infection	Decrease toxicity	1995
DaunoXome^®^ (Galen)	Daunorubicin	Liposomes	Karposi’s sarcoma	Increase site-specific delivery (tumor) and decrease toxicity	1996
DepoCyt© (Sigma-Tau)	Cytarabine	Liposomes	Lymphomatous meningitis	Increase site-specific delivery (tumor) and decrease toxicity	1996
AmBisome^®^ (Gilead Sciences)	Amphotericin B	Liposomes	Fungal and/or protozoal infections	Reduced nephrotoxicity	1997
Curosurf^®^/Poractant alpha (Chiesei farmaceutici)	Proteins SP-B and SP-C	Liposomes	Lung activator for stress disorder; pulmonary surfactant for respiratory distress syndrome	Decrease toxicity and increased delivery for smaller volume;	1999
Visudyne^®^ (Bausch and Lomb)	Verteporfin	Liposomes	Ocular histoplasmosis, myopia, decreased vision	Increase site-specific delivery (lesion vessels) photosensitive release	2000
DepoDur^®^ (Pacira Pharmaceuticals)	Morphine sulfate	Liposomes	Prolonged release	Loss of pain (post-operative)	2004
Marqibo^®^ (Onco TCS)	Vincristine	Liposomes	Acute lymphoblastic leukemia	Increase site-specific delivery (tumor) and decrease toxicity	2012
Onivyde^®^ (Merrimack)	Irinotecan	Liposomes	Pancreatic cancer	Increase site-specific delivery (tumor) and decrease toxicity	2015
Adagen^®^ (Sigma-Tau Pharmaceuticals)	Pegademase bovine	PEGylated adenosine deaminase enzyme	Immunodeficiency disease	Improve circulation time in body and decrease immunogenicity	1990
Oncaspar^®^ (Enzon Pharmaceuticals)	l-Asparaginase	PEGylated l-asparaginase	Acute lymphoblastic leukemia	Improved protein stability due PEGylation	1994
Copaxone^®^ (Teva)	Glatopa	l-Glutamate, l-alanine, l-lysine and l-tyrosine random copolymer	Multiple sclerosis	Regulation of clearance and polymer with controlled molecular weight	1996
Renagel^®^ (Sanofi)	Sevelamer hydrochloride or sevelamer carbonate	Poly(allylamine hydrochloride)	Chronic renal diseases	Increase site-specific delivery and increase in circulation time in body	2000
PegIntron^®^ (Merck)	*Interferon*-*alpha* (*IFN*-α2b)	PEGylated IFN-α2b protein	Hepatitis C	Improved protein stability due PEGylation	2001
Pegasys^®^ (Genentech)	*Interferon*-*alpha* (*IFN*-α2a)	PEGylated IFN-α2a protein	Hepatitis B and C	Improved protein stability due PEGylation	2002
Eligard^®^ (Tolmar)	Leuprolide acetate	Polymer (PLGH (poly(dl-lactide-*co* glycolide)	Prostate cancer	Prolonged drug delivery and circulation time in body	2002
Neulasta^®^ (Amgen)	PEG-filgrastim	PEGylated granulocyte colony-stimulating factor (GCSF) protein	Neutropenia induced by chemotherapy	Improved protein stability due PEGylation	2002
Somavert^®^ (Pfizer)	PEG-visomant	PEGylated HGH receptor antagonist	Acromegaly	Improved protein stability due PEGylation	2003
Macugen^®^ (Bausch & Lomb)	PEG-aptanib	PEGylated anti vascular endothelial growth factor aptamer	Macular degeneration; neovascular age-related (decreased vision)	Improved stability due PEGylation	2004
Mircera^®^ (Hoffman-La Roche)	Methoxy polyethylene glycol-epoetin beta	Chemically synthesized erythropoiesis-stimulating agent	Anemia associated with renal failure due to diseases	Improved stability due PEGylation	2007
Cimzia^®^ (UCB)	Certolizumab pegol	PEGylated antibody fragment (Certolizumab)	Crohn’s disease; rheumatoid arthritis; psoriatic arthritis and ankylosing spondylitis	Increase stability and circulation time in body	2008; 2009; 2013; 2013
Krystexxa^®^ (Horizon)	PEG-loticase	PEGylated porcine-like uricase	Chronic gout	Improved protein stability due to PEGylation	2010
Plegridy^®^ (Biogen)	*Interferon*-beta (*IFN*-β1a)	PEGylated *IFN*-β1a protein	Multiple sclerosis	Improved protein stability due to PEGylation	2015
ADYNOVATE (Baxalta)	*F*actor VIII	PEGylated factor VIII	Hemophilia	Improved protein stability due to PEGylation	2015
Rapamune^®^ (Wyeth Pharmaceuticals)	Sirolimus	Nanocrystals	Immunosuppressant	Increased bioavailability	2000
Megace ES^®^ (Par Pharmaceuticals)	Megestrol acetate	Nanocrystals	Anti-anorexic	Reduced posology	2001
Avinza^®^ (Pfizer)	Morphine sulfate	Nanocrystals	Mental stimulant	Prolonged release and increased bioavailability	2002/2015
Ritalin LA^®^ (Novartis)	Methylphenidate HCl	Nanocrystals	Mental stimulant	Increased drug loading and bioavailability	2002
Zanaflex^®^ (Acorda)	Tizanidine HCl	Nanocrystals	Muscle relaxant	Increased bioavailability and decreased posology	2002
Emend^®^ (Merck)	Aprepitant	Nanocrystals	Antiemetic drug	Increased absorption and bioavailability	2003
Vitoss^®^ (Stryker)	Calcium phosphate	Nanocrystals	Bone substitute	Mimics bone structure by cell adhesion and growth	2003
OsSatura^®^ (IsoTis Orthobiologics)	Hydroxyapatite	Nanocrystals	Bone substitute	Mimics bone structure by cell adhesion and growth	2003
Ostim^®^ (Heraseus Kulzer)	Hydroxyapatite	Nanocrystals	Bone substitute	Mimics bone structure by cell adhesion and growth	2004
Tricor^®^ (Lupin Atlantis)	Fenofibrate	Nanocrystals	Hyperlipidemia	Increased bioavailability	2004
Focalin XR^®^ (Novartis)	Dexmethylphenidate HCl	Nanocrystals	Mental stimulant	Increased bioavailability	2005
NanOss^®^ (Rti Surgical)	Hydroxypatite	Nanocrystals	Bone substitute	Mimics bone structure by cell adhesion and growth	2005
EquivaBone^®^ (Zimmer Biomet)	Hydroxypatite	Nanocrystals	Bone substitute	Mimics bone structure	2009
Invega^®^ Sustenna^®^ (Janssen Pharms)	Paliperidone palmitate	Nanocrystals	Schizophrenia schizoaffective disorder	Decreased release of poor water-soluble drugs	2009/2014
Ryanodex^®^ (Eagle Pharmaceuticals)	Dantrolene sodium	Nanocrystals	Malignant hypothermia	Allows higher administration at higher doses	2014
Estrasorb™ (Novavax)	Estradiol	Micelles	Menopause hormone therapy	Sustained release	2003
Abraxane^®^ (Celgene)	Paclitaxel (ABI-007)	Albumin-bound paclitaxel nanoparticles	Breast cancer; non-small cell lung cancer and pancreatic cancer	Increase site-specific delivery (tumor) and solubility	2005;2012;2013
INFeD^®^ (Sanofi Avertis)	Iron	Iron dextran (low MW)	Chronic kidney failure with iron deficiency	Increased dose capacity	1957
DexIron^®^/Dexferrum^®^ (Sanofi Avertis)	Iron	Iron dextran (high MW)	Chronic kidney failure with iron deficiency	Increased dose capacity	1957
Feridex^®^/Endorem^®^ (AMAG pharmaceuticals)	Superparamagnetic iron oxide nanoparticles (SPION)	SPION coated with dextran	Imaging material	Superparamagnetic character	1996/2008
Ferrlecit^®^ (Sanofi Avertis)	Sodium ferric	Sodium ferric gluconate	Chronic kidney failure with iron deficiency	Increased dose capacity	1999
Venofer^®^ (Luitpold Pharmaceuticals)	Iron oxide	Iron sucrose	Chronic kidney failure with iron deficiency	Increased dose capacity	2000
GastroMARK™; umirem^®^ (AMAG pharmaceuticals)	Superparamagnetic iron oxide nanoparticles (SPION)	SPION coated with silicone	Imaging material	Superparamagnetic character	2001/2009
Feraheme™ (AMAG pharmaceuticals)	*Ferumoxytol*-ultrasmall superparamagnetic iron oxide nanopartilces (SPION)	Ferumoxytol SPION with polyglucose sorbitol carboxymethylether	Chronic kidney failure with iron deficiency	Prolonged steady release and decreased number of doses	2009
Nanotherm^®^ (MagForce)	Iron oxide	Aminosilane-coated Iron nanoparticles	Brain tumor	Thermotherapy for destroy tumor cells or sensitized for additional therapies	2010

According to a recent review by Caster et al. [249], although few nanomedicines have been regulated by the FDA there are many initiatives that are currently in progress in terms of clinical trials suggesting many nanotechnology-based new drugs will soon be able to reach the market. Among these nanomaterials that are in phase of study, 18 are directed to chemotherapeutics; 15 are intended for antimicrobial agents; 28 are for different medical applications and psychological diseases, autoimmune conditions and many others and 30 are aimed at nucleic acid based therapies [249]. The list of nanomedicine approved by FDA classified by type of carrier/material used in preparation of the formulation is shown in Table 2.

Nanotechnology has dynamically developed in recent years, and all countries, whether developed or not, are increasing their investments in research and development in this field. However, researchers who work with practical applications of the nano-drugs deal with high levels of uncertainties, such as a framing a clear definition of these products; characterization of these nanomaterials in relation to safety and toxicity; and the lack of effective regulation. Although the list of approved nanomedicine is quite extensive, the insufficiency of specific regulatory guidelines for the development and characterization of these nanomaterials end up hampering its clinical potential [250]. The structure/function relationships of various nanomaterials, as well as their characteristics, composition and surface coating, interacts with the biological systems. In addition, it is important to evaluate the possibility of aggregate and agglomerate formation when these nanomedicines are introduced into biological systems, since they do not reflect the properties of the individual particle; this may generate different results and/or unexpected toxic effects depending on the nano-formulation [250].

The lack of standard protocols for nanomedicines characterization at physico-chemical and physiological/biological levels has often limited the efforts of many researchers to determine the toxic potential of nano-drugs in the early stages of testing, and that resulted in the failures in late-phase clinical trials. To simplify and/or shorten the approval process for nano based medicines/drugs, drug delivery system etc., a closer cooperation among regulatory agencies is warranted [204, 251].

As a strategy for the lack of regulation of nanomedicines and nano drug delivery system; the safety assessment and the toxicity and compatibility of these are performed based on the regulations used by the FDA for conventional drugs. After gaining the status of a new research drug (Investigational New Drug, IND) by the FDA, nanomedicines, nano-drug delivery systems begin the clinical trials phase to investigate their safety and efficacy in humans. These clinical trials are divided into three phases: phase 1 (mainly assesses safety); phase 2 (mainly evaluates efficacy) and phase 3 (safety, efficacy and dosage are evaluated). After approval in these three phases the IND can be filed by the FDA to request endorsement of the new nanomedicine or nano drug delivery systems. However, this approach to nanomedicine regulation has been extensively questioned [204, 246, 252].

Due to the rapid development of nanotechnology as well as its potential use of nanomedicine, a reformed and more integrated regulatory approach is urgently required. In this regard, country governments must come together to develop new protocols that must be specific and sufficiently rigorous to address any safety concerns, thus ensuring the release of safe and beneficial nanomedicine for patients [204, 252, 253].

## Future of nanomedicine and drug delivery system

The science of nanomedicine is currently among the most fascinating areas of research. A lot of research in this field in the last two decades has already led to the filling of 1500 patents and completion of several dozens of clinical trials [254]. As outlined in the various sections above, cancer appears to be the best example of diseases where both its diagnosis and therapy have benefited from nonmedical technologies. By using various types of nanoparticles for the delivery of the accurate amount of drug to the affected cells such as the cancer/tumour cells, without disturbing the physiology of the normal cells, the application of nanomedicine and nano-drug delivery system is certainly the trend that will remain to be the future arena of research and development for decades to come.

The examples of nanoparticles showed in this communications are not uniform in their size, with some truly measuring in nanometers while others are measured in sub-micrometers (over 100 nm). More research on materials with more consistent uniformity and drug loading and release capacity would be the further area of research. Considerable amount of progress in the use of metals-based nanoparticles for diagnostic purposes has also been addressed in this review. The application of these metals including gold and silver both in diagnosis and therapy is an area of research that could potentially lead to wider application of nanomedicines in the future. One major enthusiasm in this direction includes the gold-nanoparticles that appear to be well absorbed in soft tumour tissues and making the tumour susceptible to radiation (e.g., in the near infrared region) based heat therapy for selective elimination.

Despite the overwhelming understanding of the future prospect of nanomedicine and nano-drug delivery system, its real impact in healthcare system, even in cancer therapy/diagnosis, remains to be very limited. This attributes to the field being a new area of science with only two decades of real research on the subject and many key fundamental attributes still being unknown. The fundamental markers of diseased tissues including key biological markers that allow absolute targeting without altering the normal cellular process is one main future area of research. Ultimately, the application of nanomedicine will advance with our increasing knowledge of diseases at molecular level or that mirrors a nanomaterial-subcellular size comparable marker identification to open up avenues for new diagnosis/therapy. Hence, understanding the molecular signatures of disease in the future will lead to advances in nanomedicine applications. Beyond what we have outlined in this review using the known nanoprobes and nanotheragnostics products, further research would be key for the wider application of nanomedicine.

The concept of controlled release of specific drugs at the beleaguered sites, technology for the assessment of these events, drug’s effect in tissues/cellular level, as well as theoretical mathematical models of predication have not yet been perfected. Numerous studies in nanomedicine areas are centered in biomaterials and formulation studies that appear to be the initial stages of the biomedicine applications. Valuable data in potential application as drug therapeutic and diagnosis studies will come from animal studies and multidisciplinary researches that requires significant amount of time and research resources. With the growing global trend to look for more precise medicines and diagnosis, the future for a more intelligent and multi-centered approach of nanomedicine and nano-drug delivery technology looks bright.

There has been lots of enthusiasm with the simplistic view of development of nanorobots (and nanodevices) that function in tissue diagnosis and repair mechanism with full external control mechanism. This has not yet been a reality and remains a futuristic research that perhaps could be attained by mankind in the very near future. As with their benefits, however, the potential risk of nanomedicines both to humans and the environment at large require long term study too. Hence, proper impact analysis of the possible acute or chronic toxicity effects of new nanomaterials on humans and environment must be analyzed. As nanomedicines gain popularity, their affordability would be another area of research that needs more research input. Finally, the regulation of nanomedicines, as elaborated in the previous section will continue to evolve alongside the advances in nanomedicine applications.

## Conclusion

The present review discusses the recent advances in nanomedicines, including technological progresses in the delivery of old and new drugs as well as novel diagnostic methodologies. A range of nano-dimensional materials, including nanorobots and nanosensors that are applicable to diagnose, precisely deliver to targets, sense or activate materials in live system have been outlined. Initially, the use of nanotechnology was largely based on enhancing the solubility, absorption, bioavailability, and controlled-release of drugs. Even though the discovery of nanodrugs deal with high levels of uncertainties, and the discovery of pharmacologically active compounds from natural sources is not a favored option today, as compared to some 50 years ago; hence enhancing the efficacy of known natural bioactive compounds through nanotechnology has become a common feature. Good examples are the therapeutic application of nanotechnology for berberine, curcumin, ellagic acid, resveratrol, curcumin and quercetin. The efficacy of these natural products has greatly improved through the use of nanocarriers formulated with gold, silver, cadmium sulphide, and titanium dioxide polymeric nanoparticles together with solid lipid nanoparticles, crystal nanoparticles, liposomes, micelles, superparamagnetic iron oxide nanoparticles and dendrimers.

There has been a continued demand for novel natural biomaterials for their quality of being biodegradable, biocompatible, readily availability, renewable and low toxicity. Beyond identifying such polysaccharides and proteins natural biopolymers, research on making them more stable under industrial processing environment and biological matrix through techniques such as crosslinking is among the most advanced research area nowadays. Polymeric nanoparticles (nanocapsules and nanospheres) synthesized through solvent evaporation, emulsion polymerization and surfactant-free emulsion polymerization have also been widely introduced. One of the great interest in the development of nanomedicine in recent years relates to the integration of therapy and diagnosis (theranostic) as exemplified by cancer as a disease model. Good examples have been encapsulated such as, oleic acid-coated iron oxide nanoparticles for diagnostic applications through near-infrared; photodynamic detection of colorectal cancer using alginate and folic acid based chitosan nanoparticles; utilization of cathepsin B as metastatic processes fluorogenic peptide probes conjugated to glycol chitosan nanoparticles; iron oxide coated hyaluronic acid as a biopolymeric material in cancer therapy; and dextran among others.

Since the 1990s, the list of FDA-approved nanotechnology-based products and clinical trials has staggeringly increased and include synthetic polymer particles; liposome formulations; micellar nanoparticles; protein nanoparticles; nanocrystals and many others often in combination with drugs or biologics. Even though regulatory mechanisms for nanomedicines along with safety/toxicity assessments will be the subject of further development in the future, nanomedicine has already revolutionized the way we discover and administer drugs in biological systems. Thanks to advances in nanomedicine, our ability to diagnose diseases and even combining diagnosis with therapy has also became a reality.

## Abbreviations

AR: Amaranth red
CBF: ciliary beat frequency
CBZ: carbamazepine
CC: colorectal cancer
CMC: carboxymethylcellulose
Cys-MNA: ((2-amino-2-carboxyethyl) disulfanyl) nicotinic acid (Cys-MNA)
EPR: penetrability and holding
FA: folic acid-conjugated dextran
FDA: Food and Drug Administration
FeO: ferrous oxide
HA: hyaluronic acid
HDLs: high density lipoproteins
HPMC: hydroxypropylmethylcellulose
LDLs: low density lipoproteins
MR: magnetic resonance
NIR: near infrared
NP: nanoparticle
PFH: perfluorohexane
PTX: paclitaxel
RPG: repaglidine
VEGF: antivascular endothelial growth factor
VLF: venlafaxine
XG: xanthan gum
